# Single-cell genomics of single soil aggregates: methodological assessment and potential implications with a focus on nitrogen metabolism

**DOI:** 10.3389/fmicb.2025.1557188

**Published:** 2025-04-07

**Authors:** Emi Matsumura, Hiromi Kato, Shintaro Hara, Tsubasa Ohbayashi, Koji Ito, Ryo Shingubara, Tomoya Kawakami, Satoshi Mitsunobu, Tatsuya Saeki, Soichiro Tsuda, Kiwamu Minamisawa, Rota Wagai

**Affiliations:** ^1^Institute for Agro-Environmental Sciences (NIAES), National Agriculture and Food Research Organization (NARO), Tsukuba, Japan; ^2^Graduate School of Life Science, Tohoku University, Sendai, Japan; ^3^Research Center for Advanced Analysis (NAAC), National Agriculture and Food Research Organization (NARO), Sendai, Japan; ^4^Graduate School of Agriculture, Ehime University, Matsuyama, Japan; ^5^bitBiome Inc., Tokyo, Japan

**Keywords:** water-stable macroaggregates, soil microbial ecology, cell extraction method, sonication, nitrogen cycling, bacterial community compositions, functional diversity, EPS-related gene

## Abstract

Soil particles in plant rooting zones are largely clustered to form porous structural units called aggregates where highly diverse microorganisms inhabit and drive biogeochemical cycling. The complete extraction of microbial cells and DNA from soil is a substantial task as certain microorganisms exhibit strong adhesion to soil surfaces and/or inhabit deep within aggregates. However, the degree of aggregate dispersion and the efficacy of extraction have rarely been examined, and thus, adequate cell extraction methods from soil remain unclear. We aimed to develop an optimal method of cell extraction for single-cell genomics (SCG) analysis of single soil aggregates by focusing on water-stable macroaggregates (diameter: 5.6–8.2 mm) from the topsoil of cultivated Acrisol. We postulated that the extraction of microorganisms with distinct taxonomy and functions could be achieved depending on the degree of soil aggregate dispersion. To test this idea, we used six individual aggregates and performed both SCG sequencing and amplicon analysis. While both bead-vortexing and sonication dispersion techniques improved the extractability of bacterial cells compared to previous ones, the sonication technique led to more efficient dispersion and yielded a higher number and more diverse microorganisms than the bead technique. Furthermore, the analyses of nitrogen cycling and exopolysaccharides-related genes suggested that the sonication-assisted extraction led to the greater recovery of microorganisms strongly attached to soil particles and/or inhabited the aggregate subunits that were more physically stable (e.g., aggregate core). Further SCG analysis revealed that all six aggregates held intact microorganisms holding the genes (potentials) to convert nitrate into all possible nitrogen forms while some low-abundance genes showed inter-aggregate heterogeneity. Overall, all six aggregates studied showed similarities in pore characteristics, phylum-level composition, and microbial functional redundancy. Together, these results suggest that water-stable macroaggregates may act as a functional unit in soil and show potential as a useful experimental unit in soil microbial ecology. Our study also suggests that conventional methods employed for the extraction of cells and DNA may not be optimal. The findings of this study emphasize the necessity of advancing extraction methodologies to facilitate a more comprehensive understanding of microbial diversity and function in soil environments.

## 1 Introduction

Soil microorganisms inhabit a spatially heterogeneous environment, thereby driving biogeochemical cycles essential to terrestrial ecosystems. Soil structure, in particular aggregates, plays a critical role in the creation of heterogeneous microenvironments that are physically more stable habitats for microorganisms than their surrounding soil matrix (Oades and Waters, 1991; Rillig et al., 2017; Wilpiszeski et al., 2019; Kleber et al., 2021). Aggregates can be separated by sieving and form during the development of the surface soil layer in pedogenes (Oades, 1993; Totsche et al., 2018; Wagai et al., 2023; Yudina and Kuzyakov, 2023). In a conceptual model of aggregate hierarchy, it has been proposed that the small particles including microaggregates (<250 μm) are bound together by short-lived binding agents (e.g., fine roots and fungal hyphae) to form macroaggregates, defined as >250 μm diameter (Tisdall and Oades, 1982). Conversely, the microaggregates exhibit greater physical stability due to the strong binding by iron (hydr)oxide, short-range-order minerals, organo-metal complexes, and microbial debris and extracellular polymeric substances (Chenu and Cosentino, 2011; Six et al., 2004; Totsche et al., 2018). While diverse physicochemical properties of the soil within the hierarchic structure contribute to soil functions (Gupta and Germida, 2015; Hartmann and Six, 2023), fundamental questions remain regarding the interactions between soil structural heterogeneity and microbial function. For instance, although aggregates can be considered relatively discrete physical units as a part of natural soil structure, the degree to which microbial community composition varies across individual aggregates is poorly known (Kravchenko et al., 2014; Szoboszlay and Tebbe, 2021; Simon et al., 2024). Key remaining questions include the following: what determines the microbial community structure within individual aggregates, what are the taxonomic identities and genetic profiles of bacteria within these aggregates, and where are they specifically located within the soil structural matrix? Addressing these questions will require innovative methods beyond conventional bulk soil genome analysis, which implicitly assumes that soil is a homogeneous environment or that soil extraction allows unbiased recovery of microorganisms or their DNA from the heterogeneous soil microenvironment.

Advances in molecular biology have made metagenomics a powerful tool for studying the relationship between functional and taxonomic diversity in microbial communities (Tringe et al., 2005; Pan et al., 2014; Leff et al., 2015; Fierer et al., 2012b; Fierer et al., 2012a; Fierer et al., 2013). The two primary metagenomic methods are amplicon analysis and shotgun sequencing. Shotgun metagenomics, which provides both functional and taxonomic insights, has several advantages and limitations. One major advantage of shotgun metagenomics is that it allows the abundance of each gene to be associated with specific ecological processes, enabling the simultaneous examination of multiple ecosystem functions within a single soil sample (Allison and Martiny, 2008; Flinkstrom et al., 2024). This multifaceted approach recognizes the significance of ecosystem multifunctionality (Hector and Bagchi, 2007). However, shotgun metagenomics also has some limitations. For example, ribosomal protein genes are often absent from metagenome-assembled genomes (MAGs), complicating functional profiling (Mise and Iwasaki, 2022). Moreover, metagenomic sequencing struggles to achieve strain-resolved genomes (Arikawa and Hosokawa, 2023), despite the substantial functional diversity that can exist between strains (Lin et al., 2016; Krause et al., 2006; Hwangbo et al., 2016). Additionally, because shotgun sequencing data often include extracellular DNA, the presence of exogenous DNA can artificially inflate estimates of microbial diversity and genomic potential (Carini et al., 2016; Alteio et al., 2020). High-resolution genomic analyses capable of distinguishing between strains are therefore necessary. In recent years, single-cell genomics (SCG) has emerged as a complementary approach to shotgun metagenomics. This method bypasses the binning step inherent in shotgun analysis and enables the analysis of individual, intact cells (Gawad et al., 2016). Most of the single amplified genomes (SAGs) are derived from intact cells (Ide et al., 2022), allowing them to be treated as complete genomic representations of individual organisms.

By applying SCG to specific soil components, we may be able to determine the spatial distribution of functionally different bacteria, providing insights into both their habitats and taxonomic identities. For SCG application to soil, it is essential to first disperse the soil sample and separate microbial cells from soil particles. The characteristics of soil, especially clay and organic matter contents, significantly influence the efficiency of cell extraction (El Mujtar et al., 2022). The choice of dispersion method would thus be crucial in single-cell analysis, as harsher dispersion conditions can increase cell recovery but may compromise cell viability (Ouyang et al., 2021; Lee et al., 2021). To detach cells from soil particles, both physical dispersion methods (e.g., blending, sonication, vortexing, and shaking) and chemical dispersion methods (using ionic or non-ionic buffers) are commonly employed in combination (Williamson et al., 2011; Lindahl and Bakken, 1995; Courtois et al., 2001; Bakken, 1985). For small sample volumes or high-throughput analyses, shaking, vortexing (with or without beads), and sonication are frequently used (Khalili et al., 2019; Frossard et al., 2016). Sonication, also called ultrasonic treatment, applied at varying energy levels, has been used to partially or fully disperse soil aggregates. This method can reveal factors affecting soil aggregate stability through stepwise disaggregation (e.g., Asano and Wagai, 2014). The sonication approach has demonstrated a higher recovery rate for cell extraction than other methods and is effective in isolating cells (Uhlířová and Šantrůčková, 2003; Neumann et al., 2013; Frostegård et al., 1999). However, some studies have reported negative results for cell viability following sonication (Lindahl and Bakken, 1995; Khalili et al., 2019; Ellery and Schleyer, 1984). Currently, it remains uncertain what extraction technique is suitable for SCG analysis of soil samples.

The variation in the extraction techniques may have a significant impact on how we interpret soil genomic data, and this issue may be more critical when studying the soil nitrogen (N) cycle as it is tightly controlled by specific microbial functional groups that likely prefer distinct soil microenvironment (Hayatsu et al., 2008; Levy-Booth et al., 2014; Kuypers et al., 2018). For instance, the production and transformation of nitrous oxide (N₂O), a potent greenhouse gas, in the soil are known to show extremely high spatial and temporal variability (Groffman et al., 2009; Butterbach-Bahl et al., 2013), whereas denitrification including N₂O reduction generally occurs under anaerobic conditions (e.g., Schlüter et al., 2024). Recently, Mitsunobu et al. (2025) demonstrated a functional link among soil physical properties (i.e., pore network structure), O₂/N₂O concentrations, and N₂O-reducer community at a single water-stable macroaggregate scale. Specifically, the localization of N₂O reducers at the anaerobic interior of the aggregates was revealed by cryo-slicing in combination with quantitative PCR analysis. More generally, the heterogeneity of the soil microenvironment contributes to the distribution of distinct microbial community compositions. These compositions may vary according to aggregate size (Sessitsch et al., 2001; Hemkemeyer et al., 2015) and the interior and exterior of aggregates (Ranjard et al., 1998; Hattori, 1967). Thus, a better understanding of the microbial communities present within aggregates and other physically distinct soil components offers significant insights into the spatial organization of soil microbiota, which is crucial to elucidate their role in biogeochemical cycles.

A multitude of technical challenges must be overcome to examine the relationship between biophysical complexity and genomic diversity present within bulk soils. To this end, the current study aimed to characterize soil bacterial community using both metagenomic and SCG approaches by focusing on water-stable macroaggregates, the soil physical subunits that retain the intact micro-structure and microbial habitat, and can be isolated reproducibly. Specifically, we hypothesized that microorganisms having different taxonomic identities and functions can be extracted depending on the degree of soil dispersion and aggregate fragmentation. The specific objectives of this study were as follows: (i) to establish an extraction method that allows SCG analysis on single macroaggregates by comparing soil dispersion techniques and (ii) to clarify the similarity and differences in taxonomic and functional diversity of bacterial communities among the individual aggregates with a focus on N-cycle.

We intended to fully disperse soil aggregates to maximize cell extraction because some functionally important microorganisms may be localized in the interior of aggregates (e.g., N_2_O reducers; Mitsunobu et al., 2025) and/or difficult to detach from the soil due presumably to persistent biofilm formation and inhabitation to physically stable subunits (El Mujtar et al., 2022; Costa et al., 2018; Almås et al., 2005). Using the water-stable macroaggregates isolated from the cropland topsoil (light clay, Acrisol) that was previously studied (Mitsunobu et al., 2025), we optimized soil extraction method by evaluating a series of extraction and cell purification techniques by taking into account the tradeoff between aggregate dispersion and cell damage. Subsequently, we compared two dispersion techniques (bead-vortexing and sonication) and evaluated the recovery of the bacterial community based on quantitative PCR and amplicon-based bacterial composition. Finally, we characterized the N-cycle and other functional genes and taxonomic and functional diversity by performing SCG analysis of the extracted cells.

## 2 Materials and methods

### 2.1 Soil sample and incubation

Soil aggregates were collected and incubated in the same manner by Mitsunobu et al. (2025). The soil aggregates were collected from the 0- to 20-cm layer of an Acrisol that has been receiving bark compost at the long-term field trial plot of the Shizuoka Prefectural Research Institute of Agriculture and Forestry in Iwata, Shizuoka (34°45′12″N, 137°50″33E). The average annual temperatures and annual precipitation rates were 16.8°C and 1843 mm/year, respectively (Hamamatsu, 1991–2020). In the basic soil properties, bulk density was 1.2 g/cm^3^, clay was 37.7%, silt was 25.8%, sand was 36.5%, total organic carbon was 3.01%, C/N ratio was 15, and pH (H_2_O) was 7.0. We transported the soil samples to the laboratory and stored them at 4°C until wet sieving.

Water-stable soil macroaggregates were isolated by immersing the field-moist soil sample in autoclaved Milli-Q water on top of a 2-mm autoclaved sieve for 10 min, followed by manual vertical movement at the rate of 60 times over a distance of 1.5 cm for 2 min. We selected the aggregates of 5–8 mm in diameter (Supplementary Table S1) and air-dried them for 3 days under a fume hood covered with a paper towel to prevent the contamination of the dust.

We incubated the soil aggregates with artificial soil water which contained low levels of nitric acid and glucose to induce N-cycling in a chamber simulating an atmospheric environment. Using the same methods as in Mitsunobu et al. (2025), we incubated the soil aggregates with the medium simulating soil water based on Yamazaki et al. (2004) with a slight modification. The medium contains 210 μM glucose, 350 μM KNO_3_, and a solution of 410 μM NaH_2_PO_4_・2H_2_O, 160 μM Na_2_HPO_4_・12H_2_O, 84 μM MgSO_4_・7H_2_O, 200 μM CaCl_2_・2H_2_O which adjusted to pH 6.4. All media were autoclaved. We placed the soil aggregate on the autoclaved glass filter paper in a Petri dish and then dropped 1–1.5 mL of the medium per aggregate onto the paper, which allowed it to soak into the soil aggregate a little at a time without breaking the aggregate. The incubation was carried out in the dark, at 25°C and 48 h. We changed the air four times during incubation to simulate atmospheric conditions.

The weight of incubated aggregates was measured before and after the incubation. Aggregate diameter and volume were measured by taking a picture of the aggregate taken directly above using ImageJ tool. The outline of the aggregate was extracted from the image, and the Feret diameter was used as the diameter. We then calculated the aggregate volume from the diameter. Using non-incubated water-stable macroaggregates (*n* = 5), aggregate pore characteristics were assessed using X-ray micro-computed tomography (μCT). The μCT images of three of the five aggregates were obtained from a previous study (Mitsunobu et al., 2025), which gives a detailed method description. Briefly, the aggregates were scanned at beamline 20B2 of SPring-8 with a resolution of 2.71–2.72 μm per voxel. The μCT images were segmented using grayscale values to distinguish pores from the solid phase. Porosity was calculated as the ratio of pore volume to total aggregate volume. The pore depth distribution was analyzed by dividing each aggregate into three regions: 0–600 μm from the surface, 600–1,200 μm, and 1,200 μm to the center.

### 2.2 Cell extraction method development

#### 2.2.1 Pilot1. Conventional method

First, we tested the applicability of the extraction method used in previous studies that applied SCG to soils, with a slight modification (Hosokawa et al., 2022; Nishikawa et al., 2022). We first mixed 1 g bulk soil samples and a buffer (soil: buffer ratio = 1 g: 3 mL) in a 15-mL tube. The buffer we used in this study was 20 mM potassium phosphate (KPi, pH 7.5) with 0.05% Tween-80. The mixture was dispersed using a mechanical shaking technique at 120 rpm for 10 min and then allowed to stand at room temperature for 5 min; 2 mL of the supernatant was collected in a 1.5-mL tube and then centrifuged at 8,000 × g for 5 min using a benchtop centrifuge (75004251, Thermo Fisher Scientific, United States). These steps (collection and centrifugation) were repeated two times for purification. After the last mixture was centrifuged at 300 × g for 5 min, the supernatant was collected. The supernatant was stained with fluorescent dyes. LIVE/DEAD^™^ BacLight^™^ Bacterial Viability Kit (Thermo Fisher Scientific, United States) was used for estimating cell numbers; 8 μL of the supernatant was mixed with 1 μL of SYTO9 (50 μM) and 1 μL of propidium iodide (PI, 0.5 mg/μL), incubated for 10 min in the dark at room temperature and observed under a fluorescence microscope (Eclipse Ni-U, Nikon, Japan).

#### 2.2.2 Pilot2. Comparison of two aggregate dispersion techniques (bead-vortexing and ultrasonication)

We compared the following two dispersion treatments using 0.5 and 0.25 g of bulk soil (dry weight). In the bead-vortexing treatment, the incubated soil was dispersed in KPi buffer (soil: buffer ratio = 1 g: 3 mL) with sterile zirconia beads (3 mm in diameter, soil: beads ratio = 1 g: approximately 3 g, equivalent to 16 beads). The dispersion was performed by vortexing for 1 min using a small vortex mixer (N-81, Nissin, Japan). For the 0.25 g treatment, vortexing was conducted for 0.5 min, according to Whiteley et al. (2003) and Griffiths et al. (2003). For the sonication treatment, the soil plus buffer mixture prepared in the same way as for the bead-vortexing treatment was dispersed using an ultrasonic homogenizer (VP-300, TAITEC, Japan) at a low energy intensity of 30 J/ mL (15% intensity cycle of 20-s on, 20-s off), according to Neumann et al. (2013). The tip of the probe (VP-MT03) was immersed 10 mm into the soil suspension, and the sonication was done in an ice bath to reduce cell damage. The mixture after each dispersion treatment was centrifuged at 300 × g for 5 min, and the supernatant was collected.

The degree of aggregate dispersion was assessed by comparing the particle size distribution of single aggregates after the three dispersion treatments (mechanical shaking, bead-vortexing, and sonication) by laser scattering particle size distribution analyzer (LA-920, HORIBA Corporation, Japan). Three replicates (aggregates) of each treatment were performed. In other words, the volume of particles included in the distribution model on the small size side (<1.98 μm) was compared. This threshold was identified as the volume-based local minimum between the two modal peaks and located in the 1.73–1.98 μm particle size class range in the sonication and bead-vortexing dispersion samples.

We compared the cell number on LIVE/ DEAD staining in each dispersion treatment as in Pilot 1. We observed large amounts of fine soil particles, especially after the sonication dispersion (Supplementary Figure S1). We thus explored purification techniques to remove the soil particles from the suspension.

#### 2.2.3 Pilot3. Purification technique after dispersion

We first tested the density gradient method using Nycodenz (Kallmeyer et al., 2008). The supernatant was collected from 10 g and 0.5 g of incubated soil after the sonication treatment. The Nycodenz (Serumwerk Bernburg AG, Germany) with 1.3 g/mL density (60% w/v) was carefully layered under the supernatant in a ratio of 2:7 (supernatant: Nycodenz). The sample tube was centrifuged at 10,000× g for 20 min, resulting in a sharp band of bacterial cells. They were carefully collected into a new tube and then moved to microscopic observation. For 10 g of soil, more than 10^8–9^ cells/soil sample was observed. For 0.5 g of soil, however, the sharp band of bacterial cells was not observed after density separation. We thus concluded that Nycodenz purification was not suitable for single aggregates.

**Figure 1:**
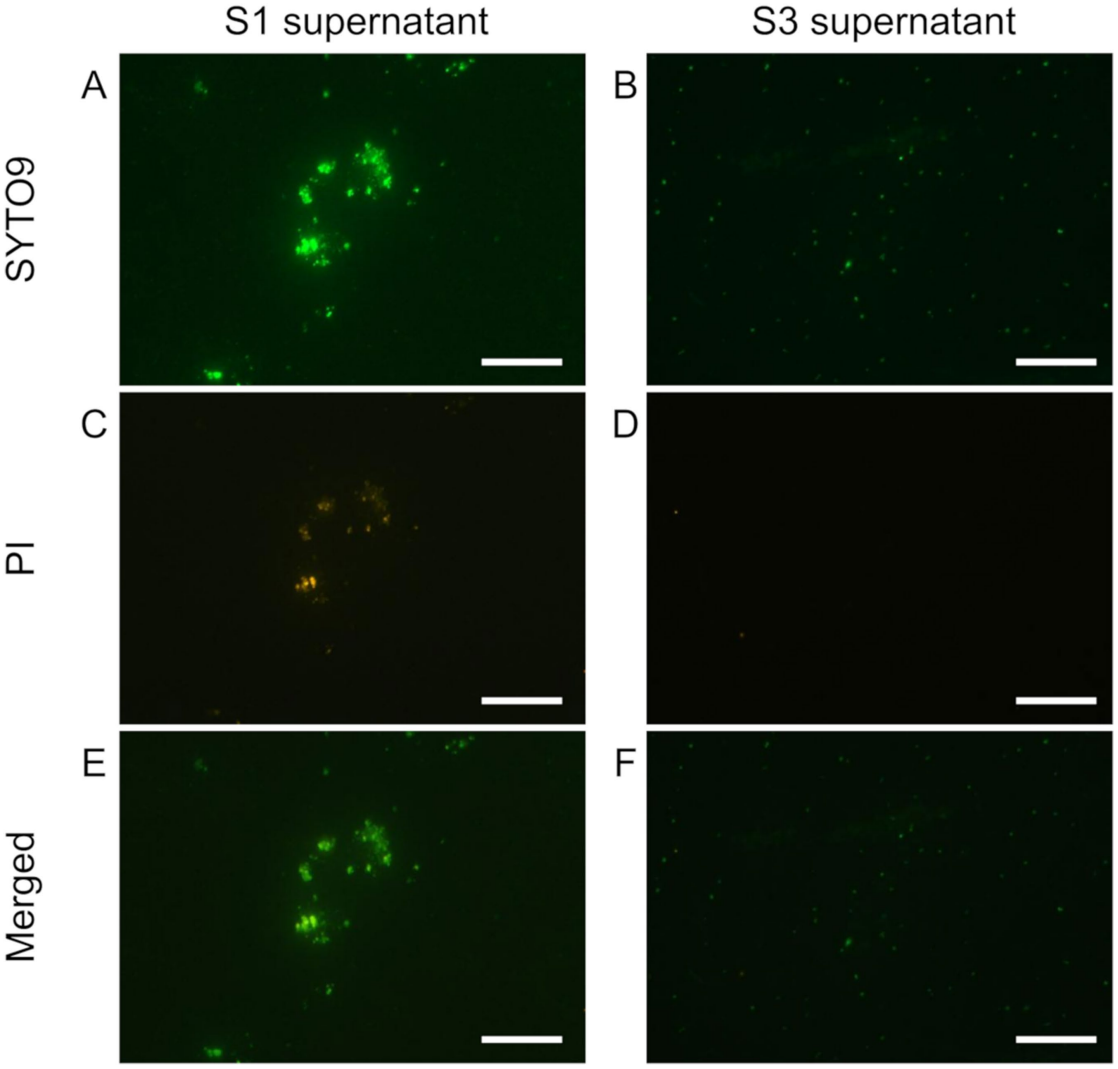
Live/dead staining of the soil supernatants obtained from repeated washing steps with sonication and dispersion. 1st supernatant (S1, in **A,C,E**) and 3rd supernatant (S3 in **B,D,F**) were stained with SYTO9 and PI. SYTO9 **(A,B)**, PI **(C,D)**, and merged **(E,F)** images are shown. The bar indicates 50 μm. S3 has fewer PI-stained particles, indicating that it was purified to cells only.

Then, we tested the sequential washing technique with 0.5 g and 0.25 g of bulk soil, following Bakken (1985). The approximately 500 μL and 250 μL of supernatant (S0) were transferred to new tubes after each dispersion treatment described above and centrifuged at 600 × g for 15 min, resulting in the first supernatant (S1). After S1 was removed, 500 μL KPi buffer was added to the residue (R1) and subjected to repeated mixing–centrifugation steps, resulting in a series of supernatants (S2 and S3) and RS3. We observed the degree of dispersion and the cell number of each supernatant (S1, S2, and S3 in Figure 1 and Supplementary Figure S1).

### 2.3 Final cell extraction method

**Figure 2:**
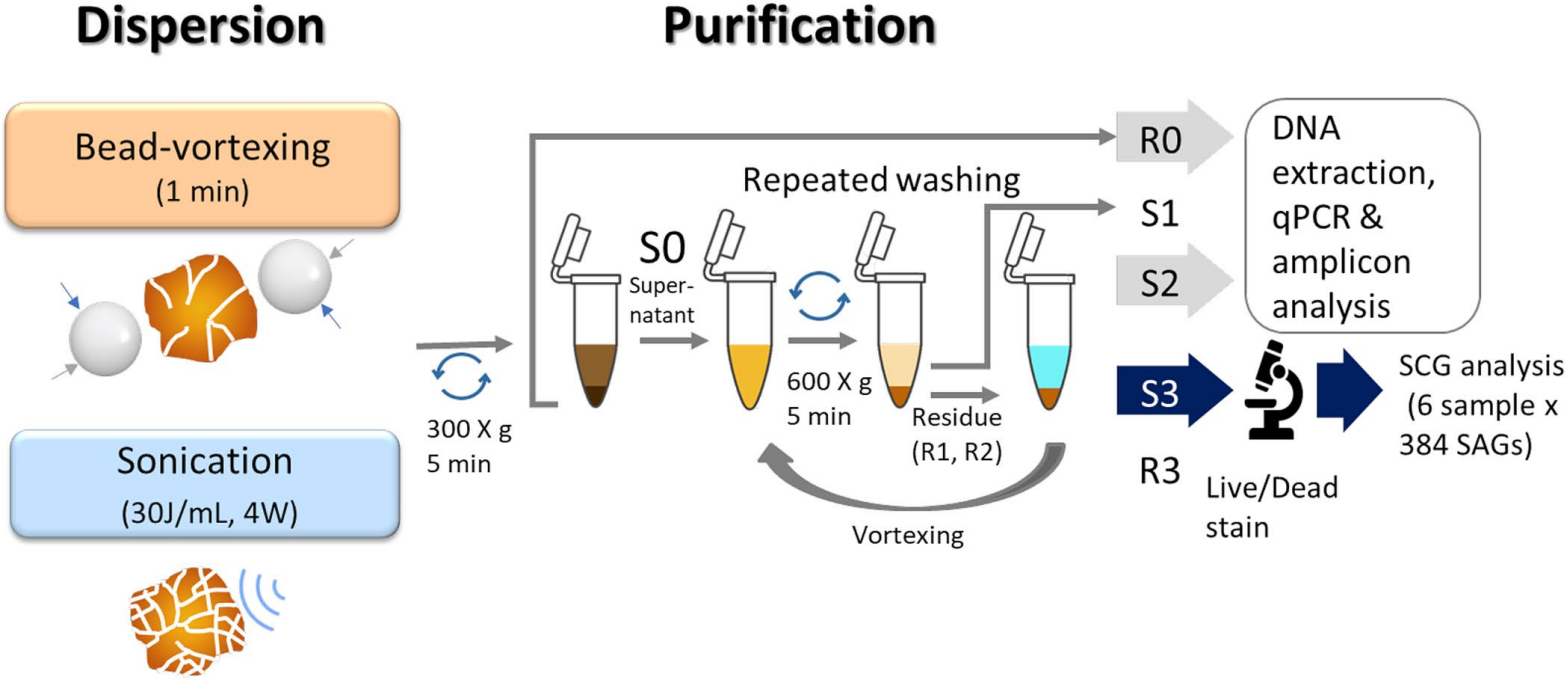
Illustration of a protocol for extraction of soil bacteria. We used the residues (R0) and the 2nd supernatant (S2) for qPCR and amplicon analysis and the 3rd supernatant (S3) for single-cell genomics analysis.

For the bead-vortexing treatment, sterile zirconia beads (3 mm diameter) in each 1.5-mL tube and vortexed for 1 min. For the sonication treatment, an incubated aggregate was filled up with KPi buffer to 4 mL in a 15-mL centrifuge tube. The mixture was dispersed using an ultrasonic homogenizer (30 J/ mL, 4 W, cycle of 20-s on, 20-s off) cooling on ice. In case a soil aggregate was above 0.1 g (dry weight), the tube was filled up to 8 mL. The mixture treated with each dispersion technique was centrifuged at 300 × g for 5 min. The approximately 200 μL in the bead-vortexing treatment or 3.8 mL in the sonication treatment of supernatant (S0) was then collected in a 1.5-mL tube. The first supernatant (S1) was removed, and the residue (R1) and new 100 μL KPi buffer were subjected to repeated mixing–centrifugation steps in the same manner as Pilot3. We proceeded with the third supernatant (S3) to the following steps. The treatment procedures are illustrated in Figure 2.

### 2.4 Single-cell genomic analysis

We used a total of six aggregates (three per dispersion treatment) for single-cell genomics. SAGs of soil microorganisms were obtained using the SAG-gel method (Chijiiwa et al., 2020; Nishikawa et al., 2022) from the third supernatant (S3) resulting from two dispersal techniques and purification (2.3). The concentration of cells was determined using LIVE/DEAD BacLight Bacterial Viability Assay and the cells were then suspended in DPBS with 1.5% low-gelling-temperature agarose (Sigma-Aldrich, MO, United States) at 1 cell/capsule. After microfluidic single-cell encapsulation in the capsules, the single-cell-encapsulating gel capsules were recovered in the aqueous phase. Then, gel capsules were immersed in Buffer D2 to denature DNA, and multiple displacement amplification was performed for 3 h using the REPLI-g Single Cell Kit (QIAGEN, Germany). After multiple displacement amplification, gel capsules were stained with SYBR Green (Thermo Fisher Scientific, United States). FACSMelody cell sorter (Becton, Dickinson and Company, United States) equipped with a 488-nm excitation laser was used to sort the gel capsules with confirmed DNA amplification into 384-well plates at 1 bead/well. Following capsule sorting, the 384-well plates were stored at −20°C. For the sequencing analysis, SAG libraries were prepared from the capsule-sorted plates using the xGen DNA Lib prep EZ UNI (Integrated DNA Technologies, Inc., United States) United States). Ligation adaptors were modified to TruSeq-Compatible Full-length Adapters UDI (Integrated DNA Technologies, Inc., United States). Each SAG library was sequenced using the DNBSEQ-G400 2 × 150 bp configuration (MGI Tech CO., Ltd., China) with the MGIEasy Universal Library Conversion Kit.

Adapter sequences and low-quality reads were eliminated from raw sequence reads of single-cell genome sequences using bbduk.sh (version 38.90) with following options (ktrim = r ref. = adapters k = 23 mink = 11 hdist = 1 tpe tbo qtrim = r trimq = 10 minlength = 40 maxns = 1 minavgquality = 15). The reads mapped to the masked human genome were eliminated using bbmap.sh (version 38.90) with following options (quickmatch fast untrim minid = 0.95 maxindel = 3 bwr = 0.16 bw = 12 minhits = 2 path = human_masked_index qtrim = rl trimq = 10). The data of the human masked index, hg19_main_mask_ribo_animal_allplant_allfungus.fa.gz, were obtained from https://zenodo.org/record/1208052#.X1hBFWf7SdY. These quality-controlled reads of single-cell genomes were assembled *de novo* into contigs using SPAdes (v3.15.2) (Bankevich et al., 2012) with the following options (--sc --careful --disable-rr --disable-gzip-output –k 2133557799127). Contigs shorter than 200 bp were excluded from the SAG assemblies. CDSs, rRNAs, and tRNAs were predicted from the SAGs using Prokka (v1.14.5) (Seemann, 2014) with the following options (--rawproduct --mincontiglen 200). The quality of the contigs was evaluated using QUAST (v5.0.2) with the default options. The completeness and contamination of SAGs were evaluated using CheckM v1.1.3 (Parks et al., 2015) in lineage workflow with the options (−r --nt) or in taxonomy workflow with the options (--nt domain Bacteria). Taxonomy identification was performed using GTDB-Tk v2.1.0 (Chaumeil et al., 2022) with default options, and GTDB release 207. The quality of each SAG was determined based on Bowers et al. (2017). SAGs that were <50% estimated completeness were considered low-quality SAGs. SAGs that had ≥50% estimated completeness and <10% estimated contamination were considered to be at least medium quality. To determine whether a SAG was high quality, in addition to having >90% estimated completeness and <5% estimated contamination, SAGs need to have 23 S, 16 S, and 5 S rRNA genes and at least 18 tRNAs present in the final assembly.

The coding sequence (CDS) and amino acid sequence of each SAG were deduced with Bakta v1.4.2 (Schwengers et al., 2021) annotation pipeline, and annotations of each CDS, were determined using eggNOG-mapper v2.1.9 (Cantalapiedra et al., 2021) with eggNOG DB version: 5.0.2 (Huerta-Cepas et al., 2019). For the SAGs of Pseudarthrobacter 87 strains that matched each other in the almost full-length 16S rRNA, the gene structures of N-cycling-related genes were compared and visualized using GenomeMatcher. The presence or absence of the following genes related to extracellular polysaccharide production (Cania et al., 2019) was then checked based on the KEGG IDs. The percentage of SAGs with at least one detected for each exopolysaccharide (EPS) gene was calculated, and the significance difference was tested using Student’s *t*-test with a threshold of 0.05. Genes encoding enzymes relevant for N-cycling were detected by diamond (Buchfink et al., 2015) blastp search of all CDSs against NCycDB (Tu et al., 2019), a curated database of N-cycling genes, with the threshold of both identity and query coverage greater than or equal to 70%. To evaluate the pathway coverage in the N-cycle (Tu et al., 2017), the abundance of functional genes annotated by NCycDB was compared between reaction paths and between aggregates. The sample number of SAGs for each aggregate was 226, 241, 269, and 206, 227, 203 SAGs, for Beads # 1–3 and Sonic # 1–3, respectively. For SAGs with *nosZ*-like genes, we examined whether *nos* accessory genes were located adjacent to the *nosZ* gene by using interProScan 5.65–97.0 (Jones et al., 2014) in EMBL-EBI with default parameters to detect protein signatures of *nosDFLY*. The structures of the *nos* gene cluster of SAGs were compared with reference genomes of related isolates or MAGs by using GenomeMatcher v3.04 (Ohtsubo et al., 2008).

### 2.5 Metagenomic analysis

We compared the supernatants and the soil residues to assess the difference in cell number and bacteria community composition to assess whether the extracted cells were representative of the soil aggregate. DNA was extracted from a total of 11 aggregate-derived R0 (0.085–0.302 g) and S2 (0.062–0.232 g) using an Extrap Soil DNA Kit Plus ver. 2 (BioDynamics Laboratory Inc., Japan) according to the manufacturer’s instructions. These 11 aggregates contained the four aggregates analyzed for SCG.

We focused on N₂O-reducing microorganisms harboring the nitrous oxide reductase gene (*nosZ*), as N₂O reduction has an important role in mitigating greenhouse gas emissions, and *nosZ*-containing microorganisms are likely to be localized in less-easily-extractable zones, i.e., the aggregate interior (Mitsunobu et al., 2025). 16S rRNA, *nosZ*-I, and *nosZ*-II amplicon sequencing and subsequent bioinformatics analysis are the same as the previous studies (Bamba et al., 2024; Hara et al., 2024). For the 16S rRNA amplicon, taxonomy was assigned to ASVs using the SINTAX algorithm (Edgar, 2016) implemented in USEARCH (v11.0.667) against the SILVA database v123 (Quast et al., 2013). The sequence reads of each sample were rarefied to 27,326 reads per sample. For *nosZ* amplicon, according to the previous study (Mitsunobu et al., 2025), the generated OTU sequences were subjected to Diamond blastx against the database of NosZ amino acid sequences retrieved from the Fungene *nosZ* repository (Fish et al., 2013). Class-level analysis was done based on NCBI taxonomy. The sequence reads of each sample were rarefied to 282 reads in *nosZ*-I and 856 reads per sample in *nosZ*-II.

### 2.6 Quantitative PCR analysis

Quantitative PCR (qPCR) assays were conducted using the fluorescent dye SYBR Green (THUNDERBIRD Next SYBR qPCR mix, TOYOBO, Japan) by a QuantStudio 3.0 real-time PCR System (Applied Biosystems/Thermo Fisher Scientific). 16S rRNA and genes coding for the two known clades of N_2_O reductase, *nosZ*-I and *nosZ*-II, were quantified using the primer pairs Bact1369F/ProK1492R (Suzuki et al., 2000) for the V3–V4 region of the 16S rRNA, nosZ-F/nosZ-R (Rich et al., 2003) for *nosZ*-I and nosZ-II-F/nosZ-II-R (Jones et al., 2013) for *nosZ*-II. The PCRs for 16S rRNA started with an initial denaturing step of 95°C for 30 s, followed by 40 cycles at 95°C for 5 s and 60°C for 30 s. Thermal cycling conditions for *nosZ*-I were initial denaturation at 95°C for 30 s, 40 cycles at 95°C for 5 s, 58°C for 30 s, and 72°C for 30 s. For *nosZ*-II, the annealing temperature was 54°C. Melting curve analyses involved a denaturing step at 95°C for 15 s, annealing at 65°C for 1 min, and melting in 0.1°C steps up to 95°C. Standard curves for each assay were generated by serial dilutions of linearized plasmids with cloned fragments of environmental DNA. Amplification efficiencies were 101.7% for 16S rRNA, 110.8% for *nosZ*-I, and 84.2% for *nosZ*-II.

### 2.7 Data analysis

The main advantage of the ASV approach is the more precise identification of microorganisms while providing a more detailed picture of the diversity in the sample relative to OTU (Wydro, 2022). Accordingly, we employed the ASV approach for 16S rRNA amplicon analysis. Unlike the 16S amplicon analysis, the other two analyses required clustering to achieve more accurate and efficient species identification. The full-length 16S rRNA sequences from the SAGs showed significant variation in sequence length. To identify sequences of the same species despite their length differences, sequences with 100% similarity were clustered into OTUs. For the *nosZ* amplicon, in addition to the high sequence length variation observed in 16S rRNA sequences of SAG, non-specific error sequences were excluded, and OTUs were defined by clustering at 99% similarity. For alpha diversity analysis, observed ASVs or OTUs, Chao1, Shannon, InvSimpson, and Faith’s phylogenetic diversity index were calculated for amplicon and SCG analysis samples. Rarefaction interpolation and extrapolation analysis of taxonomic richness (the observed ASVs) and functional richness (number of KEGG-ID and number of different kinds of N-cycling-related genes annotated by NCycDB) was performed using iNEXT v 3.0.0.packages (Hsieh et al., 2016). Furthermore, we investigated the shape of the relationship between species (OTUs based on 16S rRNA) and functional (KEGG-ID) richness to compare redundancy patterns within individual aggregates (Gamfeldt et al., 2008; Teichert et al., 2017). This relationship is expected to be linear when all species of an assemblage support singular functions, meaning that the loss of any species will produce an important and equivalent decline in functional richness. On the contrary, functionally redundant assemblages will display curvilinear relationships, i.e., saturation trends, as some functional traits are shared by multiple species.

For beta diversity analysis, a weighted UniFrac distance matrix was employed in a principal coordinate analysis (PCoA). Subsequently, a permutational multivariate analysis of variance (PERMANOVA) was conducted. The linear discriminant analysis effect size (LEfSe) method determines the features (clades, operational taxonomic units) most likely to explain differences between classes by combining standard tests of statistical significance with additional tests that encode biological consistency and effect relevance (Segata et al., 2011). Soil unit bioindicators at the phylum level were determined using LEfSe analysis with a *p*-value of 0.05.

For molecular phylogenetic analysis focused on Gemmatimonadota, as the most abundant in soil environments and potentially important role in reducing the N_2_O (Chee-Sanford et al., 2019; Jones et al., 2013; Oshiki et al., 2022), the alignment of nucleic acid was performed using MAFFT (Katoh et al., 2019). A phylogenetic tree was constructed in MEGA 7 (Kumar et al., 2016) using the maximum-likelihood method by best-fit model (Tamura–Nei model for 16S rRNA and general time reversible model for *nosZ*) selected best-fit model (Tamura et al., 2011) in bootstrap analyses based on 500 replicates.

All statistical analyses in community ecological analysis were performed in R software v4.2.1 using phyloseq v1.42.0 (McMurdie and Holmes, 2013), ggplot2 v3.5.0 (Wickham, 2011), microeco v1.4.0 (Liu et al., 2021), vegan v2.6–4 (Oksanen et al., 2013), cowplot v1.1.3 (Wilke, 2015), and ape v5.7-1 (Paradis and Schliep, 2019).

## 3 Results

In this study, we first showed that the combination of dispersion (bead-vortexing or ultrasonic) and sequential washing was the feasible method to extract intact cells from the soil aggregates for SCG analysis. Second, we performed SCG analysis using this method and showed the difference between the two dispersion techniques in their extractability of bacterial cells and DNA with respect to genome quality and their taxonomic and functional diversity. Third, by analyzing selected functional genes related to exopolysaccharides (EPS) and N-cycling, we also showed the two dispersion techniques resulted in important differences in the recovery of these genes.

### 3.1 Development and evaluation of soil bacterial extraction method

#### 3.1.1 Comparison of protocols for soil bacterial extraction

We compared previous and new methods of extracting bacterial cells from the soil aggregates for subsequent SCG analysis. Pilot1, using the previous extraction method (Hosokawa et al., 2022; Nishikawa et al., 2022), resulted in insufficient cell extraction for the studied soil (Supplementary Figure S1A). Then, we compared the two dispersion techniques (bead-vortexing and sonication treatment, Pilot2). The post-dispersion soil suspension had a bimodal particle size distribution with greater liberation of smaller sized particles (ca. 0.5 μm) at the expense of larger sized particles (ca. 15–90 μm) in the sonication treatment than the bead-vortexing treatment. In contrast, the suspension of mechanical shaking showed clearly incomplete dispersion—a much greater volume of >50 μm particles and much less volume of <1 μm particles than the other two dispersion techniques (Supplementary Figure S2). Specifically, the total volume of particles smaller than the bimodal distribution boundary value of 1.98 μm was 54.5 ± 1.6% after the sonication and 45.4 ± 9.0% after the bead-vortexing treatments (*t*-test, *p*-value = 0.1), which confirmed a greater degree of the aggregate dispersion by the sonication treatment in agreement with our visual inspection.

Aggregate pore characterization by X-ray μCT showed that the majority of the pore was present in outer zones and the interior zone (deeper than 1,200 μm from the aggregate surface to the center) accounted for only 0–13% of total pore volumes (Supplementary Figure S3) among the five randomly picked aggregates (diameter range: 4.9–7.1 mm). The pore distribution pattern suggests that outer zones are less physically stable and thus more susceptible to dispersion than the interior zones of these aggregates.

Cell counts after the two dispersion treatments were > 10^6^ (cell/ sample), which was above the minimum counts (10^6^ cell/ sample) for SCG analysis (Supplementary Figures S1B,C). In the extracted solution, we, however, observed many suspended soil particles (particularly after the sonication treatment) that would interfere with SCG analysis.

We thus conducted a sequential washing after the dispersion treatments to remove the suspended particles that would interfere with SCG analysis (Pilot3). The supernatants recovered after the first (Figures 1A,C,E) and second washings (data not shown) still contained some soil particles (microaggregates) as well as bacterial cells even after the sonication treatment. In the supernatant after the third washing, we hardly observed any aggregated particles and, based on nucleic acid staining, the number of non-bacterial particles was reduced (Figures 1E,F) while achieving the bacteria count of 10^6–7^ per sample (Supplementary Figures S1E,F). Based on these results, we adopted the soil extraction method, which combines the aggregate dispersion with the washing technique for the aggregate-scale SCG analysis (Figure 2).

#### 3.1.2 Comparison of bacteria community extracted from soil residues and supernatants

**Figure 3:**
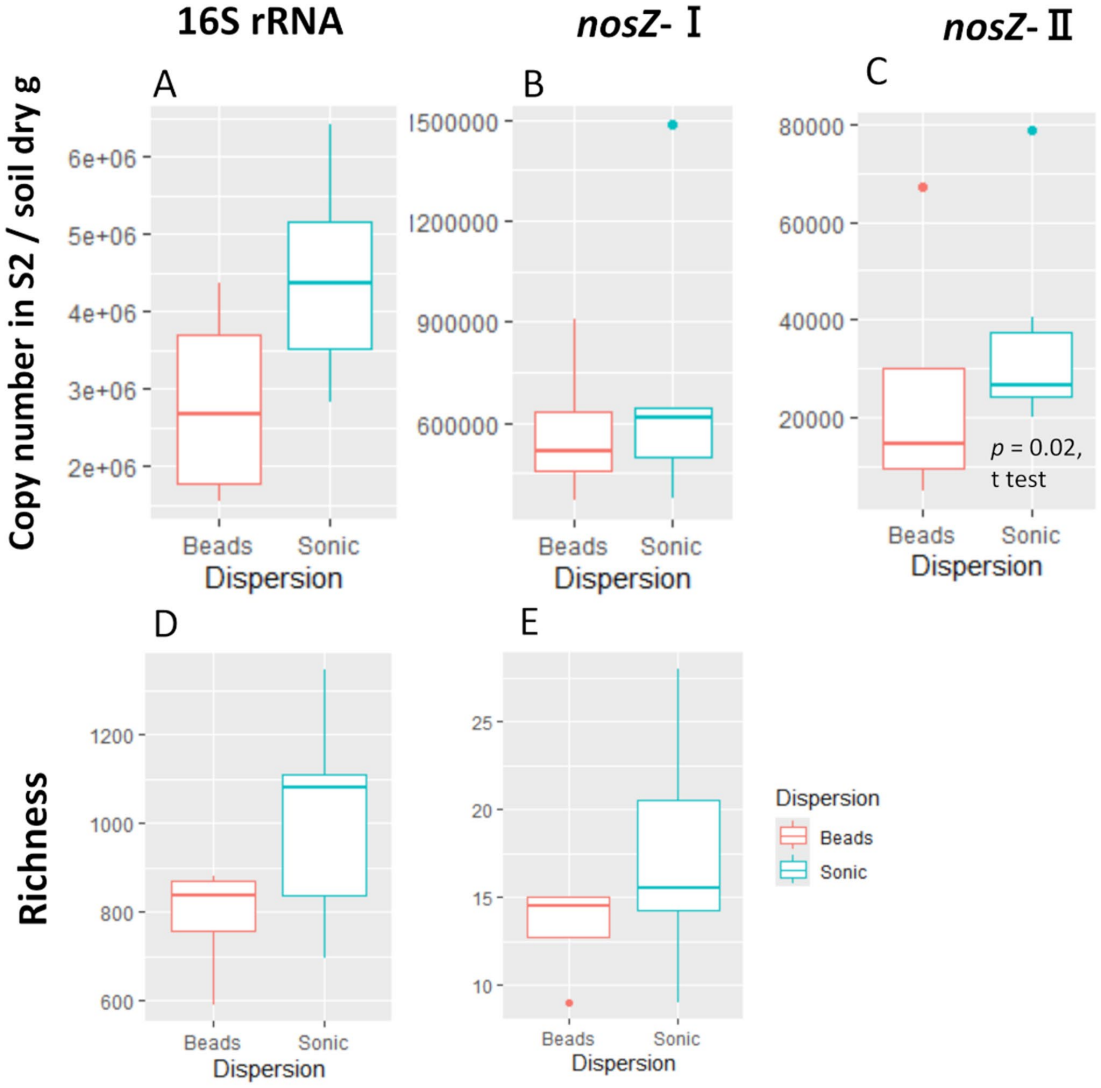
Copy number and ASV/OTU richness of the second supernatant (S2). The copy number for 16S rRNA **(A)**, *nosZ*-I **(B)**, and -II **(C)** and the richness (observed ASV/OTU) for 16S rRNA **(D)** and *nosZ* –I **(E)**. We did not perform amplicon analysis for the *nosZ*-II region due to insufficient sample volume. The significant difference was determined by *t*-test after outlier exclusion.

We compared the difference in copy number and bacteria community composition between the second supernatants (S2 in Figure 2) and the soil residues after the initial centrifugation (R0 in Figure 2) to assess the extent to which the extracted cells represent the microbiome of the single soil aggregates. The copy number in the supernatant was one to two orders of magnitude lower than that in the soil residues (Supplementary Table S1). When comparing the two dispersion techniques, the copy number of 16S rRNA, *nosZ*-I, and *nosZ*-II tended to be higher in the sonication than the bead-vortexing treatment while the significant difference was detected only for *nosZ*-II (Figure 3C, *t*-test, *p*-value = 0.02).

The amplicon analysis of individual aggregates showed that the S2/R0 ratio of ASV for 16S rRNA was 0.43 on average (range: 0.31–0.57), that for *nosZ*-I OTU was 0.24 (0.10–0.41) (Supplementary Table S1). On average in each aggregate, 53% (range: 30–70%) and 34% (14–57%) of ASVs for 16S rRNA and OTUs for *nosZ*-I in S2 were shared between R0 and S2 of total ASVs or OTUs. The Shannon diversity index of the S2 community (16S rRNA) was higher in the sonication (5.68 ± 0.29) than the bead-vortexing treatment (4.70 ± 0.17) (Supplementary Table S1, *t*-test, *p*-value = 0.01).

**Figure 4:**
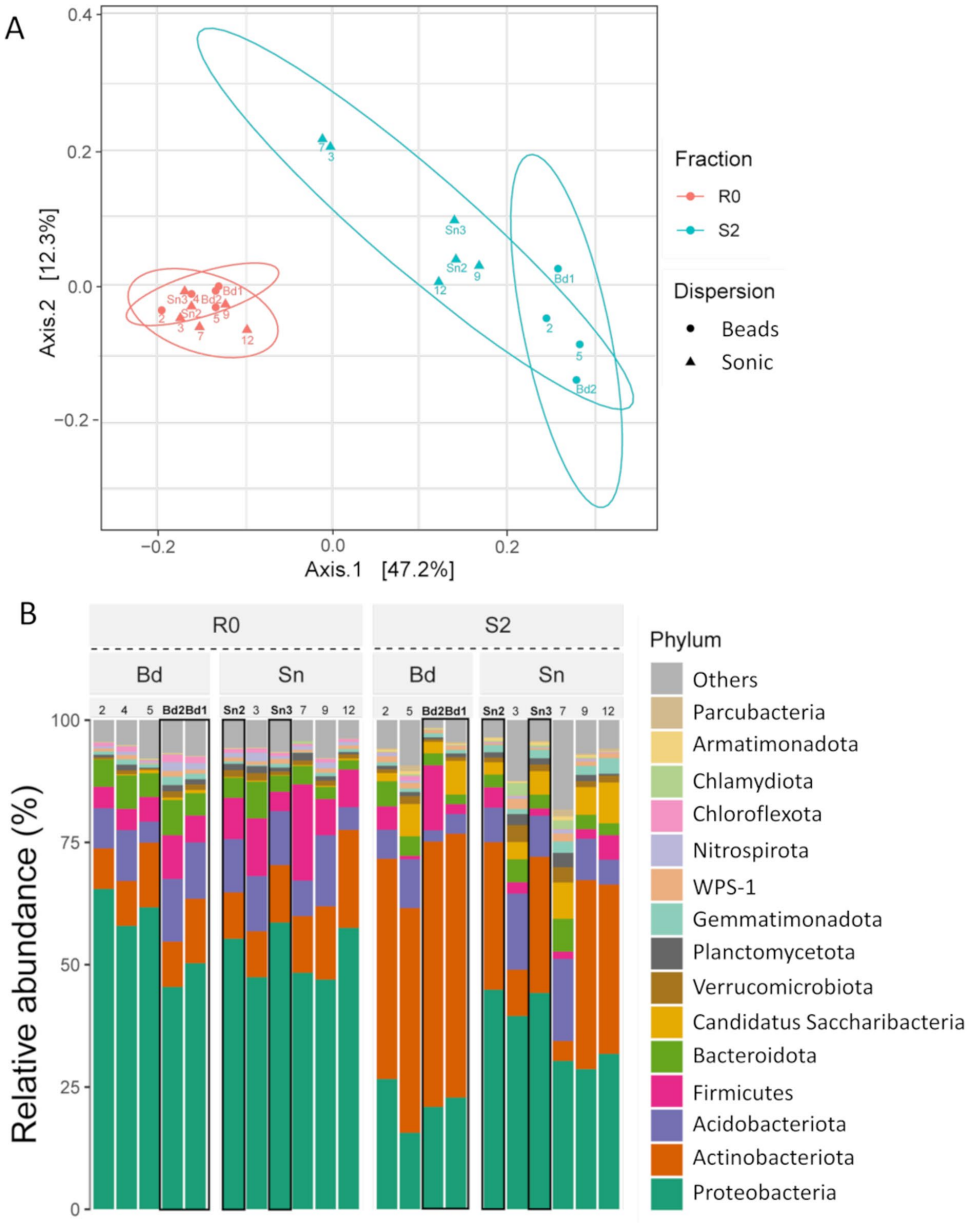
Community differences are shown by a PCoA plot of the weighted UniFrac distance matrix **(A)** and a bar chart at the phylum level **(B)** across the different fractions and dispersion. R0, residue; S2, second supernatant; Bd, after the bead-vortexing treatment; Sn, after the sonication treatment. The numbers indicate the aggregate sample number. Sn2-3 and Bd2-3 Sn3 highlighted in bold with black border indicate the sonication and bead-vortexing treatment sample in the SCG analysis.

The difference in bacterial community composition between the supernatant and the residue as well as between the two dispersion treatments was shown by PCoA of the weighted UniFrac distance based on the 16S rRNA region (PERMANOVA, fraction: *r*^2^ = 0.40, *F* = 14.8, *p*-value = 0.001, dispersion: *r*^2^ = 0.065, *F* = 2.4, *p*-value = 0.047, Figure 4A). The distance between S2 and R0 in the sonication treatment (0.31 ± 0.02) was significantly smaller (*t*-test, *p*-value < 0.001) than that in the bead-vortexing treatment (0.36 ± 0.03). In contrast to 16S rRNA, no significant difference between the two soil fractions and two dispersion treatments was found for *nosZ*-I and *nosZ*-II regions (Supplementary Figures S4A,C). We noted that *Bradyrhizobium* in *nosZ*-I and *Cloacibacterium* and *Runella* (both Bacteroidota) in *nosZ*-II appeared to be higher in the sonication than the bead-vortexing treatment (Supplementary Figures S4B,D). The characteristic phylum in S2 shown by the LEfSe analysis was Actinobacteriota, Parcubacteria for the bead-vortexing treatment and Armatimonadota, Bacteroidota, Candidatus_Saccharibacteria, and Gemmatimonadota for the sonication treatment (Supplementary Table S2).

### 3.2 Profiles of single amplified genomes extracted from single aggregates

#### 3.2.1 SAGs data quality

We evaluated basic genomic information including its quality based on the 2,304 SAGs analyzed. Of all SAGs, 2 SAGs were classified as high-quality (HQ), 241 SAGs as medium-quality (MQ), and 2047 SAGs as low-quality (LQ) genomes (Table 1). The remaining 14 SAGs had a contamination rate of 10–34%, and most of them were Actinobacteriota. The average total length was 3.72, 2.52, and 0.91 Mb; the average N50 was 101, 60, and 25 Kb; and the average number of tRNAs were 39.5, 27.9, and 10.6, for HQ, MQ, LQ, respectively. When comparing the two dispersion techniques, SAGs of HQ and MQ tended to be higher in the bead-vortexing treatment than in the sonication treatment with an average of 15.5 and 5.4%, respectively. Similar trends were also observed for the completeness, the number of tRNA, and total CDS, while no significant difference was shown for N50 (Table 1; Supplementary Figure S5). When analyzing after excluding the most dominant Actinobacteriota SAGs, all indexes tended to diverge to a greater extent between the aggregates than between the dispersion treatments (Supplementary Figure S5).

**Table 1:** Basic genomic information and quality, which are the evaluation indices for single-cell analysis.

	Beads #1	Beads #2	Beads #3	Sonic #1	Sonic #2	Sonic #3
**Reads stats**						
Number of SAGs	384	384	384	384	384	384
Total reads (Mreads)	471	444	488	357	457	389
Total length (Mbp)	70,700	66,500	73,200	53,600	68,600	58,400
Clean reads (Mreads)	440	435	480	335	446	381
Clean length (Mbp)	65,900	65,000	71,900	50,100	66,800	56,900
**Quality stats**						
High-quality SAGs	0	1	1	0	0	0
Medium-quality SAGs	34	74	71	7	24	31
Low-quality SAGs	350	301	311	377	359	349
Contaminated SAGs	0	8	1	0	1	4
**Novelty score**						
N/A	183	155	141	240	189	211
High novelty (>50%)	126	153	168	116	133	123
Low novelty (≤50%)	75	68	74	28	61	46
**Quality (average)**						
Completeness (%)	21.2	28.2	29.1	15.4	21.8	20.3
Number of contig	683	564	736	631	659	588
Total length	1.06E+06	1.31E+06	1.37E+06	8.26E+05	1.00E+06	9.85E+05
N50	2.18E+04	4.31E+04	3.97E+04	1.46E+04	2.77E+04	3.05E+04
Number of CDSs	1,071	1,291	1,334	861	964	957
Number of tRNA	11.6	15.5	15.7	9.1	11.8	11.2

#### 3.2.2 Taxonomic and functional diversity

**Figure 5:**
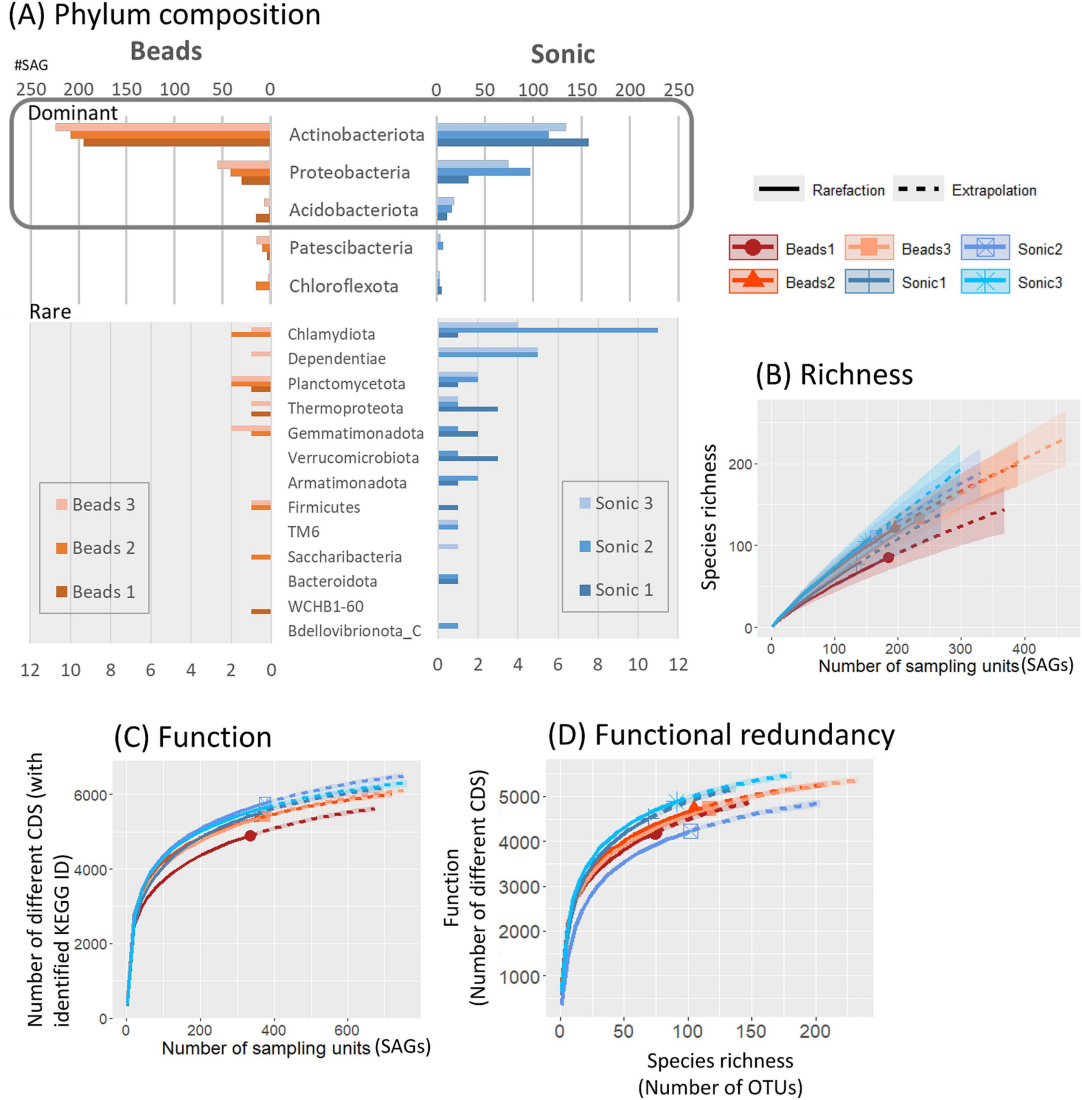
Taxonomic and functional diversity of SAGs. Taxonomic composition of SAGs, annotated based on the GTDB at the phylum level, is presented for all SAGs **(A)**. Rarefaction curves illustrate alpha diversity, including species richness based on 16S rRNA gene sequences **(B)**, functional diversity **(C)**, and functional redundancy **(D)**. Distribution of SAG counts at the genus level for the three most abundant genera within each phylum is detailed in Supplementary Figure S6.

Based on phylogenetic annotation based on the GTDB, Actinobacteriota (66.0%, 1,034/1,566 SAGs identified to the phylum level) was the most frequent phyla (Figure 5A). When comparing the two dispersion techniques among SAGs identified by phylum level, the average number of phyla detected was 12.6 in the sonication and 9.3 in the bead-vortexing treatment. The first dominant Actinobacteriota was significantly more frequent after the bead-vortexing treatment (209.0 ± 11.9 SAGs) than after the sonication (135.7 ± 16.8 SAGs) (*t*-test, *p*-value = 0.007). In contrast, the second dominant Proteobacteria and the third dominant Acidobacteriota tended to be higher after the sonication (Figure 5; Supplementary Figure S6). The results indicated that the cell extraction with the sonication dispersion allowed the detection of a wider range of phylum than that with the bead-vortexing dispersion technique. We calculated Faith’s phylogenetic diversity using the full-length 16S rRNA data. The phylogenetic diversity appeared to be higher after the sonication treatment (175.1 ± 15.4) than the bead-vortexing treatment (155.6 ± 14.7) but without statistically significant difference (*t*-test, *p*-value = 0.2). A similar trend was also observed in the rarefaction curve of ASV (Figure 5B).

We also assessed the functional diversity of the SAGs. When comparing the two dispersion techniques among SAGs, the number of different CDS based on KEGG-ID tended to be higher in the sonication than the bead-vortexing treatment (Figure 5C). The comparison of the number of functional genes with species richness showed that total number of functional genes was equally high (4,000–5,000) with similar plateau shapes for all the aggregates, suggesting high bacterial functional redundancy across the six single aggregates (Figure 5D). However, the inter-aggregate variation became noticeable when focusing on N-cycling genes (Supplementary Figure S7A), especially on specific processes (Supplementary Figures S7B–D) (see Section 3.3.2).

#### 3.2.3 Genetic variation in the comparative arrangement of gene clusters within a species

**Figure 6:**
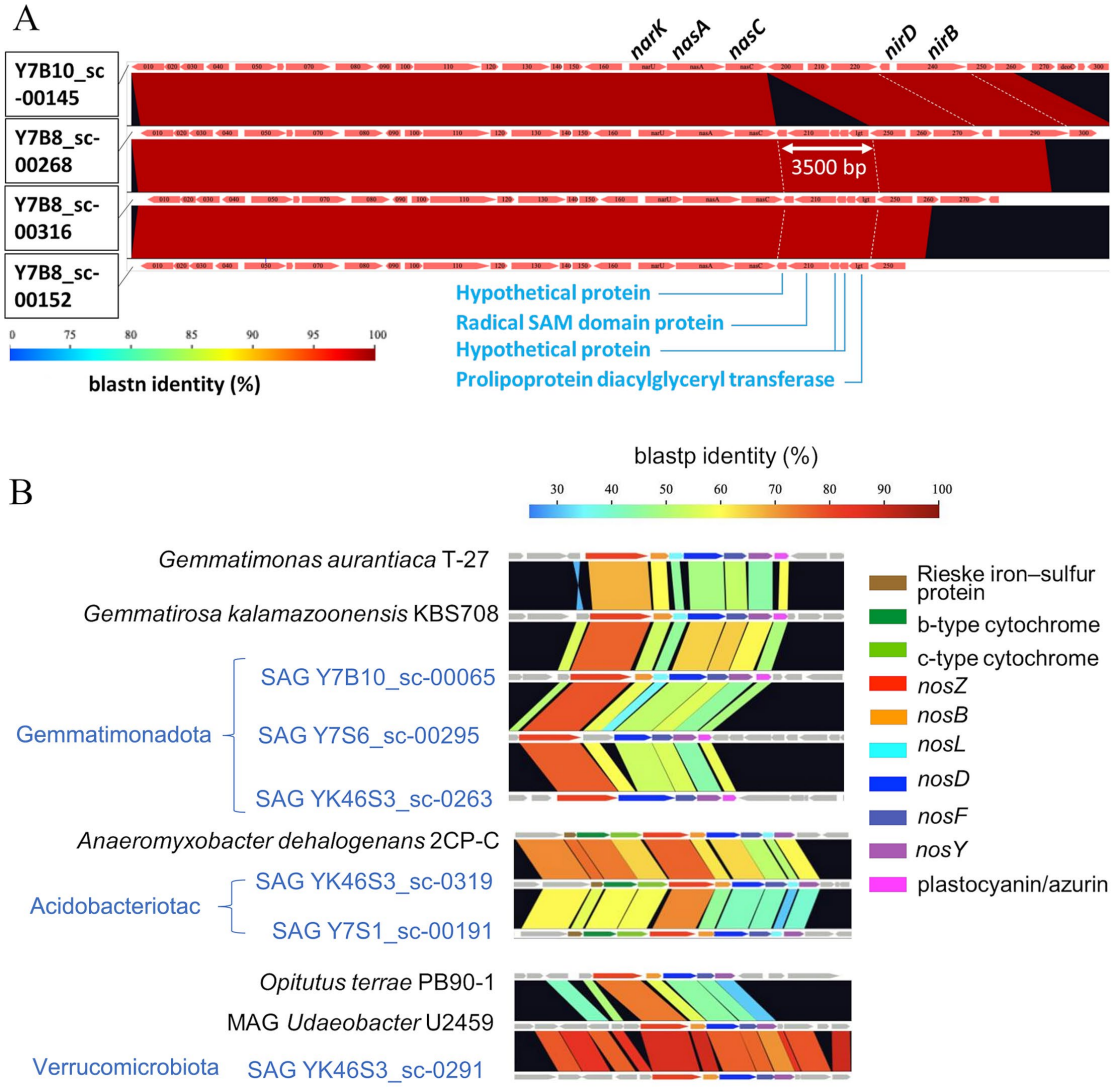
Comparative arrangement of gene cluster among SAGs. **(A)** Cluster of the assimilatory nitrate reductase and nitrite reductase cluster among four SAGs belonging to Pseudarthrobacter. **(B)** Cluster for *nos* between SAGs (blue letter) and reference strains (black letter). Color coding represents % homology based on nucleotide sequences of genes calculated by GenomeMatcher (Ohtsubo et al., 2008).

One of the advantages of single-cell analysis over MAGs is its capability of detecting the differences among the genes from closely related strains. We analyzed the SAGs of Pseudarthrobacter 87 strains that matched each other in almost full-length 16S rRNA and compared the contigs in which the assimilatory nitrate reductase and nitrite reductase clusters were located at loci of Y7B10_sc-00145, Y7B8_sc-00268, Y7B8_sc-00316, and Y7B8_sc-00152 (Figure 6A). The results showed overall very high homology, except for a loss of approximately 3,500 bp in Y7B10_sc-00145. In Y7B10_sc-00145, a region of approximately 3,500 bp encoding the radical SAM domain protein gene and the prolipoprotein diacylglyceryl transferase gene is missing, while it was conserved in the other three SAGs.

### 3.3 Functional gene analysis of single amplified genomes

The results above (3.2.2) showed that the dominant phyla were the same between the two dispersion treatments while the sonication treatment obtained higher functional diversity. Then, we carried out a more detailed profiling of functional genes that are related to soil aggregation and N-cycling.

#### 3.3.1 Comparison of SAGs harboring EPS-related genes

We investigated a total of 242 SAGs of middle and high quality for the presence of exopolysaccharide (EPS)-related genes as EPS plays a major role in cell attachment to the soil surface and aggregate formation (Wagai et al., 2023). A total of 242 SAGs of MQ and HQ were investigated for the presence of EPS-related genes (Supplementary Figure S8). The proportion of SAGs harboring the polysaccharide export outer membrane protein gene (*wza*, KO no K0991) was significantly lower in the bead-vortexing treatment than the sonication treatment, 6.1% ± 3.1% and 33.7% ± 8.5%, respectively. In addition, 36 of 61 SAGs in the sonication treatment and 16 of 181 SAGs in the bead-vortexing treatment belonged to actinomycetes, but none harbored the *wza* gene. In contrast, many SAGs (86.2% ± 12.2% in the sonication and 72.2% ± 25.5% in the bead-vortexing treatment) in bacteria other than actinomycetes harbored the *wza* gene. The same trend was observed for LptB2FGC lipopolysaccharide export complex permease gene (*lptF*, KO no. K07091), LptB2FGC lipopolysaccharide export permease gene (*lptG*, KO no. K11720), LptB2FGC lipopolysaccharide export complex inner membrane protein gene (*lptC*, KO no. K11719), and capsular polysaccharide export system permease gene (*kpsE*, KO no. K10107) (Supplementary Figure S8).

#### 3.3.2 Nitrogen-cycling genes

**Figure 7:**
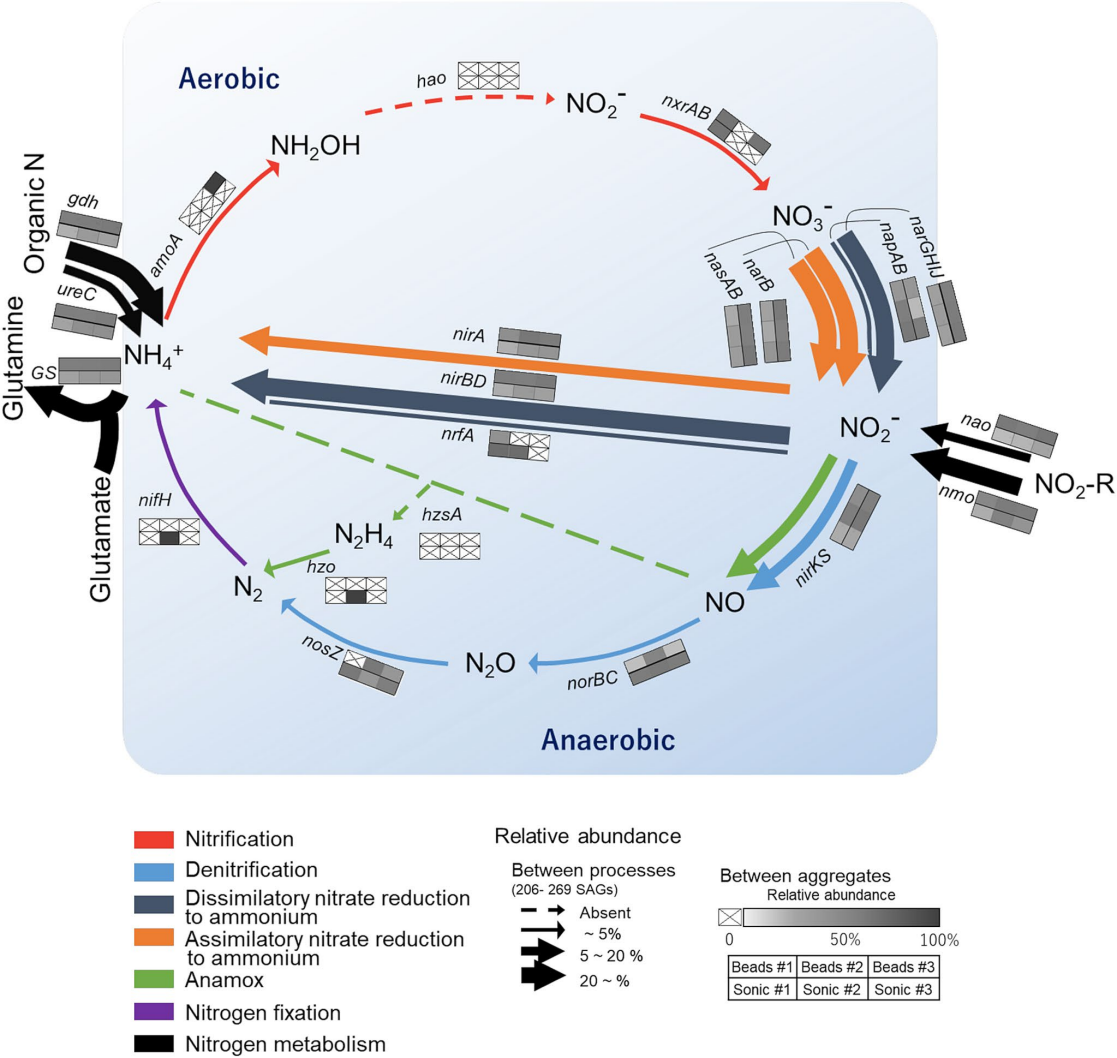
Overview of nitrogen (N) cycling genes in the single soil aggregates isolated from the studied soil. The colors of the arrows correspond to specific N transformation processes. Their thickness represents the relative abundance of SAGs harboring the functional genes corresponding to the specific N processes (calculated for each aggregate based on the total number of SAGs and then averaged for the six aggregates). The six-cell boxes with a heat map represent the SAG count in each aggregate relative to the total count of SAGs harboring the corresponding functional genes. Darker colors in the boxes show higher abundances and a cross indicates below the detection limit. The general structure of the N cycling schema following Tu et al. (2017).

We then narrowed down the functional analysis to N-cycling genes and assessed how the frequency of major N-cycling genes CDS (annotated by NCycDB) differed among the reaction paths (arrows, Figure 7) and the six soil aggregates (six-cell boxes with heatmap, Figure 7). Relatively abundant genes (>20%, bold arrows) were *nirBD* related to NO_2_^−^ → NH_4_^+^ and *narB*, *nasAB*, and *narGHIJ* related to NO_3_^−^ → NO_2_^−^. In addition, *nmo* and *gdh* genes that are related to organic nitrogen degradation and a *GS* gene involved in glutamine synthesis were abundant. On the other hand, functional genes related to nitrification, nitrogen fixation, and anammox were little detected. Low-abundance genes (<5%, thin arrow) were *nosZ*, *nrfA*, *norBC,* and *napAB*. When comparing among the aggregates, the high-abundance genes were typically found in the aggregates isolated after the bead-vortexing dispersion treatment (the upper three cells in each box, Figure 7), whereas the low-abundance genes tended to be found in the aggregates under the sonication dispersion treatment. A rarefaction estimates CDS richness tended to indicate the detection of more diverse functional genes by the sonication dispersion except for assimilatory nitrate reduction to ammonium (ANRA)-related genes (Supplementary Figure S7).

We also assessed whether each taxon (phylum) differed in the diversity of functional gene arrangements involved in the N-cycling. Actinobacteriota, the most dominant phylum, harbored multiple genes related to dissimilatory nitrate reduction to ammonium (DNRA), denitrification (nitrate and nitrite reduction), and ANRA within single cells except for Thermoleophilia (class) that harbored few N-cycling genes. Proteobacteria, the second dominant phylum, also harbored the genes for multiple N-cycling pathways. In contrast with Actinobacteriota, many classes of Proteobacteria harbored genes for denitrification-specific processes, such as *nirKS* and *nosZ*-I. The only SAGs with genes involved in nitrification belonged to archaea (Nitrososphaeraceae).

Homology search of CDSs of all SAGs by blastp against NcycDB identified NosZ-like CDSs from 15 SAGs (Table 2); 11 out of these 15 SAGs were taxonomically assigned by Gtdbtk: 5 SAGs from Acidobacteriota, 3 from Proteobacteria, 2 from Gemmatimonadota, and 1 from Verrucomicrobiota. Although the rest of the four SAGs were unclassified by Gtdbtk, their NosZ-like CDSs showed homology to inferred NosZ of Bacteroidota, Proteobacteria, and Gemmatimonadota. The 16S rRNA and *nosZ* classifications did not match for two SAGs from the bead-vortexing treatment that were not unclassified by Gtdbtk. Comparing the results of the single-cell and amplicon analyses by focusing on Gemmatimonadota, the sequences of *nosZ* and of 16S rRNA from SCG were closer to the dominant ASV of Gemmatimonadota identified by the amplicon analyses of both *nosZ* and 16S rRNA than any of the known isolated strains in Gemmatimonadota (Supplementary Figure S9).

**Table 2:** Taxonomy of *nosZ*-harboring SAGs.

Aggregate sample ID	Gtdbtk.taxonomy	16S rRNA	*nosZ* clade		NCBI blastp result		
				Description	Scientific name	Query cover	Per. Ident
Beads #2	Unclassified bacteria	*Arthrobacter*	II	Sec-dependent nitrous oxide reductase	*Flavisolibacter nicotianae*	0.96	0.97
	g_*AG11* (Gemmatimonadota)	*Gemmatimonas*	II	Sec-dependent nitrous oxide reductase	Gemmatimonadaceae bacterium	0.97	0.82
	g_*Massilia* (Proteobacteria)	*Massilia*	I	TAT-dependent nitrous oxide reductase	*Massilia agilis*	1	0.86
	f_*Fen-336* (Acidobacteriota)	ND	II	Sec-dependent nitrous oxide reductase	*Vicinamibacteria bacterium*	0.86	0.97
Beads #3	g_*Massilia* (Proteobacteria)	*Massilia*	I	TAT-dependent nitrous oxide reductase	*Massilia agilis*	1	0.92
	Unclassified bacteria	Micrococcales	I	TPA: TAT-dependent nitrous oxide reductase	*Alcaligenes faecalis*	1	1.00
Sonic #1	g_*QHWT01* (Acidobacteriota)	ND	II	Sec-dependent nitrous oxide reductase	*Acidobacteriota bacterium*	0.97	0.80
	g_*AG2* (Gemmatimonadota)	Gemmatimonadaceae	II	Sec-dependent nitrous oxide reductase	*Gemmatimonadota bacterium*	0.98	0.76
	g_*AV55* (Verrucomicrobiota)	ND	II	Sec-dependent nitrous oxide reductase	*Chthoniobacterales bacterium*	0.96	0.87
	g_*QHWT01* (Acidobacteriota)	o:Subgroup_6	II	Sec-dependent nitrous oxide reductase	*Acidobacteriota bacterium*	0.99	0.80
Sonic #2	g_*PSRF01* (Acidobacteriota)	ND	II	Sec-dependent nitrous oxide reductase	*Pyrinomonadaceae bacterium*	0.99	0.87
	Unclassified Bacteria	ND	I	TAT-dependent nitrous oxide reductase	*Microvirga vignae*	0.99	0.85
Sonic #3	g_*Hyphomicrobium*_A (Proteobacteria)	*Hyphomicrobium*	I	TAT-dependent nitrous oxide reductase	*Microvirga vignae*	0.99	0.83
	Unclassified Archaea	ND	II	TPA: Sec-dependent nitrous oxide reductase	*Gemmatimonadota bacterium*	0.97	0.76
	g_*12-FULL-67-14b* (Acidobacteriota)	ND	II	Sec-dependent nitrous oxide reductase	*Acidobacteriota bacterium*	1	0.87

Furthermore, we examined nos accessory genes located in the *nosZ*-flanking region of SAGs from Acidobacteriota, Gemmatimonadota, and Verrucomicrobiota, the phyla known to be difficult to culture. Blastp against NCBI and InterProScan analysis detected CDSs with homology to *nosDFY*, important for the formation of the active site of *nosZ*, and other *nos* accessory genes in their *nosZ*-flanking regions. Comparative analysis of *nosZ*-flanking regions revealed that the gene arrangement of the *nos* gene cluster was highly conserved among the SAGs and reference genomes (Figure 6B).

## 4 Discussion

### 4.1 Optimal extraction methods for SCG and general characteristics of extracted bacteria

We were able to obtain genomic information based on SCG analysis from single soil aggregates for the first time by optimizing the extraction method after a series of pilot tests assessing aggregate dispersion techniques (zirconia bead-vortexing and sonication) as well as the purification of dispersed soil suspension. In theory, a high recovery of bacterial cells from soil is achievable by sufficient, if not complete, dispersion of the aggregates and detachment of cells from soil surfaces while minimizing the physical damage to the cells. Our results suggested that the ultrasonic dispersion was more effective than the bead-vortexing for the recovery of bacterial cells and that of particular bacterial groups (especially *nosZ*-harboring bacteria in Figure 3C). Below, we first discuss the methodological aspects in terms of the representativeness of the number and composition of bacteria recovered.

A fundamental question when applying cell or DNA extraction techniques to soil is the extent to which the bacteria extracted represent the whole microbiome in the soil sample (Pathan et al., 2021; Wydro, 2022). To a limited extent, we assessed this by comparing the extracted bacterial genes between the soil residue obtained after the soil dispersion/centrifugation (R0) and the second supernatant of the soil suspension (S2) because the third supernatant (S3) was used for the SCG analysis and thus unavailable (Figure 2). We found that the 40% of ASVs in S2, based on 16S rRNA amplicon analysis, was common between S2 and R0 despite that the number of bacteria in the supernatant was two orders of magnitude lower than the residue (Supplementary Table S1). These results suggest that the microbial community in the third supernatant used for SCG analysis is likely to be not drastically different from the community present in the bulk sample (i.e., individual macroaggregates). While the assessment of the bacteria extraction efficiency in soil is rather rare in the literature, our results are generally consistent with the previous study which examined the bacteria recovery from pasture soils in New Zealand. Highton et al. (2023) used an extraction method comparable to ours and compared bulk soils and their water extracts. After the soil dispersion in water using a blender followed by low-speed centrifugation and washing, they used the epifluorescence microscopy-based (EFM) quantification method and showed that the number of extracted cells ranged from 3.3 to 9.4% of the bulk soil. In comparison, our current study showed that the copy numbers of S2 relative to that of R0 for 16S rRNA, *nosZ*-I, and -II were 5.2, 4.1, and 9.4%, respectively, using the qPCR method (Supplementary Table S1). To the extent that the copy number of the 16S rRNA gene correlates with the number of cells detected by EFM quantification methods (Deng et al., 2019), the bacteria recoveries in our study were comparable to those of Highton et al. (2023). Using 16S rRNA amplicon analysis, they further showed that the bacterial community in the extracts and bulk soils shared roughly half of the ASVs and the only major difference at the family level was the greater recovery of Bacillaceae (Firmicutes) in the bulk soil. In our study, the S2/R0 ratio for species richness of 16S rRNA and *nosZ*-I was 38.9 and 17.9%, respectively (Supplementary Table S1), suggesting that roughly a quarter of the bulk soil bacteria community was extracted.

A tradeoff is likely to be present between dispersion-assisted cell extraction and physical damage to bacterial cells during the dispersion, especially for strongly aggregated soils. In fact, we found that greater dispersion by the sonication treatment led to the recovery of more diverse bacteria while their SAGs were, on average, lower in quality (Table 1; Supplementary Figure S5). Genome quality is strongly affected by DNA damage caused by physical and chemical processes taking place during the sonication treatment. The sonication treatment was able to disrupt the aggregates of ca. 20–100 μm diameter sizes compared to the bead-vortexing treatment (Supplementary Figure S2). As soil bacterial community composition can be significantly different among particle/aggregate size fractions (Biesgen et al., 2020; Ranjard et al., 1998), the greater abundance and diversity of bacteria released by the sonication (Figures 3, 5) may be attributable to those associated with the 20–100 μm aggregates. The proportion of the total SAGs higher than the medium quality accounted for on average 16% (bead-vortexing treatment) and 5% (sonication treatment). The sonication-assisted extraction allowed the detection of *nos* and its surrounding genes even from low-quality category of SAGs (Figure 6B). Furthermore, the sonication treatment led to higher recovery and diversity of DNA (Figures 3, 4A) and greater diversity of SAGs including their functional genes (Figures 5A–C) than the bead-vortexing treatment. Thus, our sonication-assisted extraction method was more effective in characterizing the bacterial community present in the soil aggregates than the extraction with the bead-vortexing dispersion. While the cavitation effect of ultrasound by sonication has been demonstrated to disrupt cell structure and associated chemical effects including the generation of free radicals can damage DNA (Li et al., 2018; Lv et al., 2019; Miller et al., 1991; Oyane et al., 2009), these negative effects may not be significant.

The current extraction method possibly led to a higher recovery of soil bacterial community than previous soil SCG studies because no other studies used sonication to disperse soil aggregates. At the same time, the sonication-assisted extraction used in the current study likely led to greater degrees of cell damage—medium- and high-quality SAGs accounted for 5% (sonication treatment) and 16% (bead-vortexing treatment) of total SAGs. In comparison, Nishikawa et al. (2022) performed a mixing (details not reported) plus washing method with high-speed centrifugation for the extraction and showed that 20% of the total SAGs were in medium- and high-quality SAGs. Aoki et al. (2022), using a mixing pretreatment (details unknown) followed by density gradient centrifugation (Nycodenz), yielded on average 41% medium- and high-quality SAGs. These previous studies showed higher proportions of medium- and high-quality SAGs than the current study, which likely reflects the fact that their soil samples are typically poorly aggregated (beach, desert, mangrove, and paddy soils). In our study, the dispersion using the conventional method was insufficient (Supplementary Figures S1A, S2). For a further study targeting the bacteria in aggregates and well-aggregated soils (e.g., high in soil C, clay, and/or metal oxides), it is important to compare different methods such as the sequential washing/sonication method (Hattori, 1967; Kohno and Hashimoto, 2008) and the sequential dispersion/density gradient centrifugation method (Nadeem et al., 2013).

The physical stability of soil aggregate may exert a primary control on cell extraction efficiency given the levels of dispersive energy required for effective aggregate disruption. This hypothesis appears reasonable as cell detachment from soil surfaces is likely to be severely impeded when their habitats (e.g., pores) are present in aggregate interiors and not fully exposed to extracting solution. The aggregates studied here are relatively stable as our Acrisol is relatively high in clay and iron oxide contents (Mitsunobu et al., 2025). In these agricultural topsoils that contain significant amounts of water-stable aggregates, it would be important to sufficiently disperse aggregates for the extraction of cells that reasonably represent the soil bacterial community.

### 4.2 Greater recovery of “strongly attached bacteria” by sonication-assisted extraction

The two aggregate dispersion techniques used in this study clearly affected the recovery of soil bacteria. The supernatant after the sonication treatment showed the bacterial community composition more similar to the residual soil microbiome than those after the bead-vortexing treatment (Figure 4). The 16S rRNA amplicon sequencing results at the phylum level also showed the superiority of the sonication dispersion treatment compared to the bead-vortexing treatment but with a few exceptions. The Actinobacteriota detected after the bead-vortexing treatment was higher in relative abundance than the sonication treatment based on both the amplicon analysis and SAGs (Figure 4B; Supplementary Table S2). This result may be explained by the tendency that Actinobacteriota are easily dispersible (Ouyang et al., 2021) and tolerant to physical stress (Schimel et al., 2007). In contrast, the Bacteroidota were most abundant after the sonication treatment (LEfSe analysis in Supplementary Table S2). Several genera of Bacteroidota are considered “hard-to-extract” taxa due presumably to their strong binding to the surfaces of soil organic and mineral particles (Ouyang et al., 2021).

The following three lines of results suggest that the sonication treatment preferentially released microbial cells that are more strongly attached to soil particle surface and/or present in the interior of aggregates than the bead-vortexing treatment. First, the sonication treatment liberated more *nosZ*-harboring bacteria cells and N_2_O-related functional genes than the bead-vortexing treatment. Specifically, a larger number of the cells containing *nosZ*-I and *nosZ*-II were detected after the sonication treatment (Figures 3B,C) and the S2/ R0 ratio of *nosZ*-II (12%) was particularly higher after the sonication treatment than the bead treatment (1%). In addition, the sonication treatment led to a significantly higher abundance of Bacteroidota and Gemmatimonadota (Supplementary Table S2), the main phylum possessing *nosZ*-II (Hallin et al., 2018). Our SCG analysis further showed that Proteobacteria and Acidobacteriota, which detected *nosZ* in this study, were more diverse with the sonication treatment (Figure 5). Furthermore, the relative abundance of *nosZ* key denitrifying functional genes was higher in the sonication treatment (Figure 7).

Second, the possible localization of the extracted cells in the original aggregates can be inferred by comparing the current results with the previous study where the single aggregates (approximately 6 mm in diameter) isolated from the same bulk soil and incubated in the same way were sliced from the top to the center at approximately 300 μm intervals followed by the amplicon sequencing of each slice (Mitsunobu et al., 2025). We found that two *nos*Z sequences, detected from two SAGs (YK46S3_sc-0263 belonging to Gemmatimonadota and YK46S3_sc-0319 belonging to Acidobacteriota), had 99–100% homology to the *nosZ* OTUs isolated from the aggregate interior in the previous study (Mitsunobu et al., 2025). These OTUs were mainly detected in the interior region of the aggregates (deeper than approximately 1,200 μm), whereas some were present at 300–900 μm depth from the surface (Supplementary Figure S10). This comparison therefore supports the idea that the sonication dispersion liberated the microbial cells located in the interior as well as its exterior of the aggregate.

Third, the analysis of EPS-related genes implies the preferential extraction of the cells strongly attached to the soil surface and/or reside in physically stable subunits of the aggregates. The strongly attached bacteria are difficult to extract for two reasons. First, they are strongly attached to the surface of the soil solid phase using sticky compounds such as extracellular polymeric substances (Wagai et al., 2023), or they selectively reside in subunits of the studied macroaggregates that are difficult to break up (e.g., physically stable microaggregates present within water-stable macroaggregates, Six et al., 2004; Yudina et al., 2022). Given that extracellular polymeric substances act as a major binding agent for soil aggregation (Chenu and Cosentino, 2011; Costa et al., 2018; Oades and Waters, 1991), the strongly attached bacteria are likely associated with physically stable subunits of the incubated macroaggregates. We, therefore, hypothesized if the sonication treatment causes more dispersion of physically stable aggregate subunits, the bacteria harboring EPS-related genes are released more after the sonication than the bead-vortexing treatment. The comparison of the functional genes related to the production of EPS (Cania et al., 2019) between the two dispersion treatments revealed that, among the nine EPS genes detected, three of them were significantly higher in the sonication treatment (*p*-value < 0.05) while none was higher in the bead-vortexing treatment (Supplementary Figure S8). These differences likely resulted from non-Actinobacteriota because (i) 66% of the bacteria extracted by SCG (Figure 5A) and 83% of the high-quality bacteria belong to Actinobacteriota (Supplementary Figure S5), and (ii) the Actinobacteriota was characterized by a lower number of EPS genes (see Section 3.3.1), whereas these numbers were higher for the non-Actinobacteriota that were more extracted from the sonication treatment (see Section 3.3.1).

There are two possible explanations for the higher abundance of Actinobacteriota under the bead-vortexing treatment. First, Actinobacteriota extracted from the sonication treatment were more easily damaged than those from the bead-vortexing treatment. Second, the relative proportion of Actinobacteriota in the extracted cells was lower in the sonication treatment because the stronger aggregate dispersion by sonication released a greater number of the bacteria that were strongly attached to soil particles and/or resided in more stable microaggregates, thereby diluting the Actinobacteriota population. As a further result of supporting the second, Gram-positive bacteria having physically tough membranes (Schimel et al., 2007) such as Actinobacteriota, Firmicutes, and Saccharibacteria were more abundant in the bead-vortexing treatment. In contrast, Gram-negative bacteria such as Proteobacteria, Acidobacteriota, Dependentiae, Verrucomicrobiota, and Bacteroidota were more abundant in the sonication treatment (Figure 5A).

The observed differences in recovered bacteria between the two aggregate dispersion treatments may give important insights into the habitat and ecology of N_2_O-reducing bacteria. Nadeem et al. (2013) distinguished two types of habitats for denitrifying microorganisms based on their attachment strength to soil particles using the sequential dispersion/density gradient centrifugation method. For a sandy loam grassland soil, the authors showed that strongly attached cells produced less N_2_O than loosely attached cells, and the reduced N_2_O production by the strongly-attached cells was at least partially attributable to greater N_2_O reduction (higher activity of N_2_O reductase, *nosZ*). Thus, the strongly attached cells were presumed to be localized at “inner” habitats such as crevices and cavities of the soil particles and/or present as persistent biofilms that are more resistant to physical dispersion (Nadeem et al., 2013). The sonication treatment in our study dispersed single aggregates more effectively into finer subunits than the bead-vortexing treatment did (Supplementary Figure S2) and released more *nosZ*-harboring bacteria (Figure 3), implying that *nosZ*-harboring bacteria may be localized more in the aggregate subunits that are physically stable (e.g., the aggregate interiors where pore was much less abundant, Supplementary Figure S3) against the bead-vortexing and/or present in the cell attachment mode that was more susceptible to the fine air bubbles released by the sonication treatment than the bead-vortexing.

### 4.3 Characteristics of functional genes isolated from extracted intact cells

We were able to draw three additional inferences that were not obtainable by the metagenomics approach. For the most dominant species among the most extractable phylum, Actinobacteriota, we found inter-individual variation in the arrangement of N-cycling genes (syntenic block) within the same species. Comparison of genes around *nasC* in 4 SAGs with 100% 16S rRNA sequence match and higher (medium) genomic quality (Figure 6A) showed that one SAG lacked the 3,500 bp encoding five genes, whereas others did not. These results indicate that, even among very closely related SAGs, where the full 16S rRNA length is perfectly matched, genetic variations due to sequence deletions (or insertions) were present. Such deletions would be difficult to detect in MAGs constructed from shotgun metagenomic sequences as the sequences before and after the deletion are almost perfectly matched. In other words, the current study showed evidence of the variation in functional gene arrangement within one species, Actinobacteriota (i.e., even at the population level) for the first time in the soil environment.

Analysis of *nosZ*-flanking regions suggested that the intact cell-derived SAGs we obtained were most likely from a potentially functional *nosZ*-harboring bacteria. The genetic arrangement including the *nos* cluster was similar to that of known closely related species that are shown to have *nosZ* gene expression (Figure 6B). In particular, *Gemmatimonas aurantiaca* T-27 (Chee-Sanford et al., 2019; Park et al., 2017) has been studied in detail for N_2_O reduction. Three SAGs belonging to *Gemmatimonas* (esp. SAG of Y7B10_sc-00065) showed quite similar arrangement in the *nos* operon, indicating that the obtained SAGs are from a potentially functional *nosZ*-harboring bacteria. Therefore, not only did they retain the *nosZ*, but they were a potentially functional *nosZ*-harboring bacteria because the *nosZ* was detected as part of the *nos* operon.

The comparison of the results from the single-cell and amplicon analyses by focusing on Gemmatimonadota revealed that the sequences of *nosZ* and 16S rRNA from SCG were closer to the dominant ASV of Gemmatimonadota identified by the amplicon analyses of both *nosZ* and 16S rRNA than any of the known isolated strains in Gemmatimonadota (Supplementary Figure S9 for *nosZ* and 16S rRNA). The current study thus showed that single-cell genomics of single soil aggregates has the potential to provide genomic information on microorganisms that are closely related to those dominant in the soil aggregates based on the amplicon analysis.

### 4.4 Insights from single aggregate analyses

The rationale underlying our focus on individual soil aggregates was to achieve a more refined understanding of the taxonomic diversity and functional roles of microbial communities within intact soil microhabitats, without assuming homogeneity of bulk soil samples. We compared taxonomic and functional diversity among 6 individual aggregates based on SAG data and 11 aggregates based on amplicon data. Our results showed both uniqueness and similarity in microbial diversity and selected functional gene profiles among the studied individual aggregates.

We detected some ASVs unique to individual aggregates. The mean species accumulation plot showed the increase in observed ASVs with the increase in aggregates, at least, up to eight aggregates despite that all aggregates were incubated under the same condition before the DNA extraction (Supplementary Figure S11A). By comparing individual aggregates and the homogenized bulk soil from the same set of soil cores, Simon et al. (2024) showed that five aggregates captured higher diversity than the bulk and the ASVs unique to these aggregates accounted for 20% of total ASVs obtained. Their results indicate that these rare species were heterogeneously distributed on a small scale (Simon et al., 2024). Similarly, we found that 27% of bacterial ASVs were unique to 1 of the 11 aggregates (Supplementary Figure S11B). However, on average, the unique ASVs made up only 0.5% of the total bacterial abundances (Supplementary Figure S11B).

The comparison of N-cycling genes also showed some variations among the six aggregates. The gene families involved in anammox, nitrification, nitrogen fixation, denitrification, and DNRA, were low abundant. Two of the functional genes were not detected in any aggregates (*hao*, *hzsA*). These genes showed high inter-aggregate variations. Three factors may be considered to account for the high variability of the undetected and low-frequency genes. First, nitrification may be limited due to the low oxygen condition shown in our incubated aggregates (Mitsunobu et al., 2025) and the low concentration of ammonium relative to nitrate in our incubation. Second, *nifH*, *hao*, *nosZ*, and *hzo* are often regarded as low-abundant gene families despite their important roles in N-cycling (Kuypers et al., 2018; Tu et al., 2017). Detection of low-abundance genes is more difficult and subject to more errors. Third, only a small portion of the entire microbial community in each aggregate was extractable, and the strongly attached cells were likely to be less represented as discussed in Sections 4.1 and 4.2. For example, *nosZ*-harboring bacteria, which showed increasing trend from the outer surface toward aggregate core based on qPCR (Mitsunobu et al., 2025), were undetectable in one of the three replicates in the bead-vortexing treatment (Figure 7). In contrast, they were detected from all three replicates of the sonication treatment which led to more effective dispersion of the aggregates (Supplementary Figure S2). Furthermore, more diverse N-cycling genes (especially denitrification and DNRA) tended to be extracted after the sonication than the bead-vortexing treatment (Supplementary Figure S7), confirming the importance of the dispersion method (Discussion 4.2.). The heatmap of the relative gene abundance for each dispersion method (three boxes aligned side by side in Figure 7 box) showed some uniqueness among the individual aggregates. The variation among the triplicates was large for *nxrAB*, *nrfA*, *nosZ* (to a limited extent, *napAB*, *nirA*, *nirKS*) in both dispersion methods, for *amoA* (to a limited extent, *norBC*) in the bead-vortexing treatment only, and for *nifH* (to a limited extent, *narB*, *nasAB*, *nao*, *nmo*) in the sonication treatment. These inter-aggregate variations in the N-cycling genes may suggest the presence of unique microbial community structures in different aggregates (Szoboszlay and Tebbe, 2021).

The current study, nevertheless, revealed overall similarity in the bacterial community structure and their potential functions among the six aggregates, implying that single water-stable macroaggregates may represent microbially and biophysically stable units in soil. While the current study is limited in the number of cells analyzed by SCG (approximately 400 per aggregate) and the extraction was conducted after the laboratory incubation (as opposed to freshly isolated from the soil), the three lines of evidence support this view. First, the inter-aggregate variations in the number of bacteria were relatively small both in the residues (R0) and the supernatant (S2) (quantitative PCR, Figure 3). The variation of species richness in each fraction was also small (R0 in Supplementary Table S1; S2 in Figure 3, S3 in Figure 5B) although the effect of the two dispersion treatments was present as discussed above (Section 4.2). Second, when we sum up the ASVs between the residue and supernatant (R0 and S2), more than 60% of ASVs were common across the 11 aggregates. In addition, the three most dominant phyla were also similar among the 10 aggregates in the R0 fraction and six aggregates in the S3 fraction (Figures 4B, 5A). Third, the bacterial functional diversity and redundancy were high in a similar degree among the six single aggregates as indicated by the comparison between the number of functional genes and SAGs (Figure 5C) and OTUs (Figure 5D). While such high functional redundancy of soil microbial community has been shown at bulk soil scales (Chen et al., 2021; Louca et al., 2018), our study further showed that the high redundancy is maintained even at the individual aggregate scale (Figure 5). Fourth, we found rather similar relative abundance of the N-cycling genes across the six aggregates (Figure 7). Of the seven major pathways we analyzed, ANRA, DNRA and organic N metabolism had the highest gene abundance as depicted in the arrow thickness (Figure 7). Our results at the aggregate level are consistent with the FACE grassland study (Tu et al., 2017), which also showed Actinobacteriota as the main phylum and organic N metabolism and nitrate reduction as the two major N transformation pathways at the bulk soil level, presumably because microorganisms gain energy and nutrients by these two processes (Condron et al., 2010; Moreno-Vivián et al., 1999). Furthermore, we found that the genes associated with denitrification, ANRA, DNRA, and N metabolism showed high relative abundance in all six aggregates (see 3×2 box next to each arrow, Figure 7).

In particular, all six aggregates contained microorganisms with genes enabling the conversion of nitrate into all possible nitrogen forms, suggesting that aggregates act as a minimal functional unit for N-cycling in soil. Similarly, Simon et al. (2024) suggested that soil aggregates may act as functional units of soil organic matter turnover based on the correlations among microbial community composition, organic matter content, and its recycling status among the intact aggregates (approximately 2 mm in diameter). Similarly high functional redundancy among the aggregates shown in our study further highlights the potential value of soil aggregate as a basic experimental unit to examine microbial diversity-function relationship as aggregates largely maintain the physical, chemical, and microbial condition of *in situ* soils. Our results pose a question regarding the efficacy of contemporary cell extraction techniques, such as bead-beating or vortexing, in soils that exhibit strong aggregation due to their suboptimal recovery of cells and DNA. It is, thus, imperative to advance extraction methodologies that will facilitate a more comprehensive understanding of microbial diversity and functioning within soil environments.

## Data Availability

The datasets, sequence raw data of SAGs and amplicon sequence data for this study can be found in the the DDBJ. All data were registered using BioProject PRJDB19546. The names of the repository/repositories and accession number(s) can be found in the article/Supplementary material.
