# Supplementary material for: Single-cell genomics of single soil aggregates: methodological assessment and potential implications with a focus on nitrogen metabolism
Source: Front Microbiol. 2025 Apr 7;16:1557188. doi: 10.3389/fmicb.2025.1557188 (PMC12010503; doi:10.3389/fmicb.2025.1557188)
Supplement: Supplementary file 1 [file Data_Sheet_1.PDF]

Fig. S1

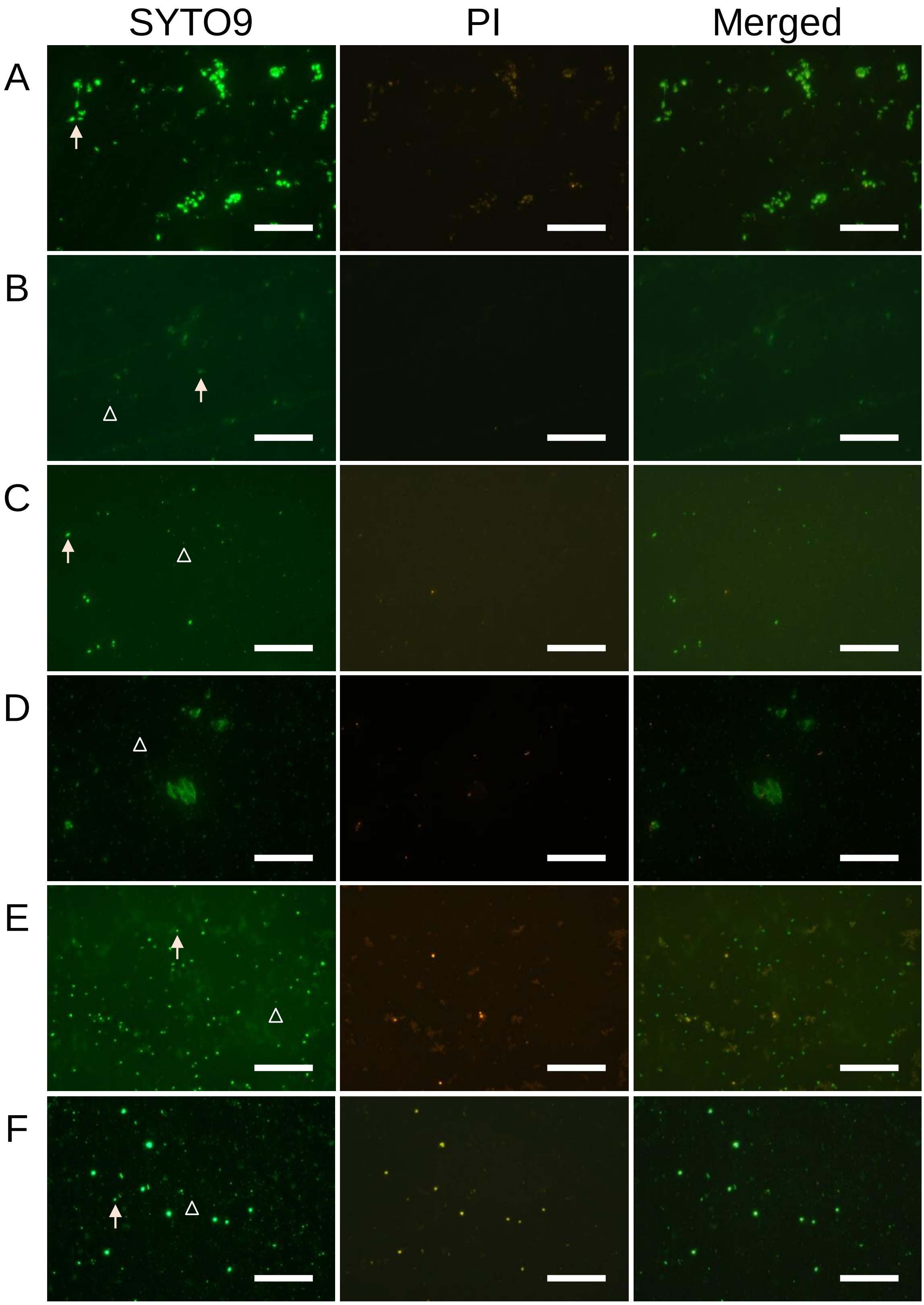

Fig. S1 Live/dead staining of the soil supernatant with conventional methods (A), with Sonic (S0, B) or Beads (S0, C), with Nycodenz purification (D), and with Sonic (S3, E) and Beads (S3, F). These samples were stained with SYTO9 and PI. SYTO9, PI and merged images were shown. Bar: 50 µm. Bacteria or soil particles were determined by the presence or absence of Brownian motion. For example, a cell similar in size to the particle indicated by the white triangle was counted as a single bacterium, whereas a cell similar in size to the particle indicated by the arrow was not counted as a single bacterium.

Fig. S2

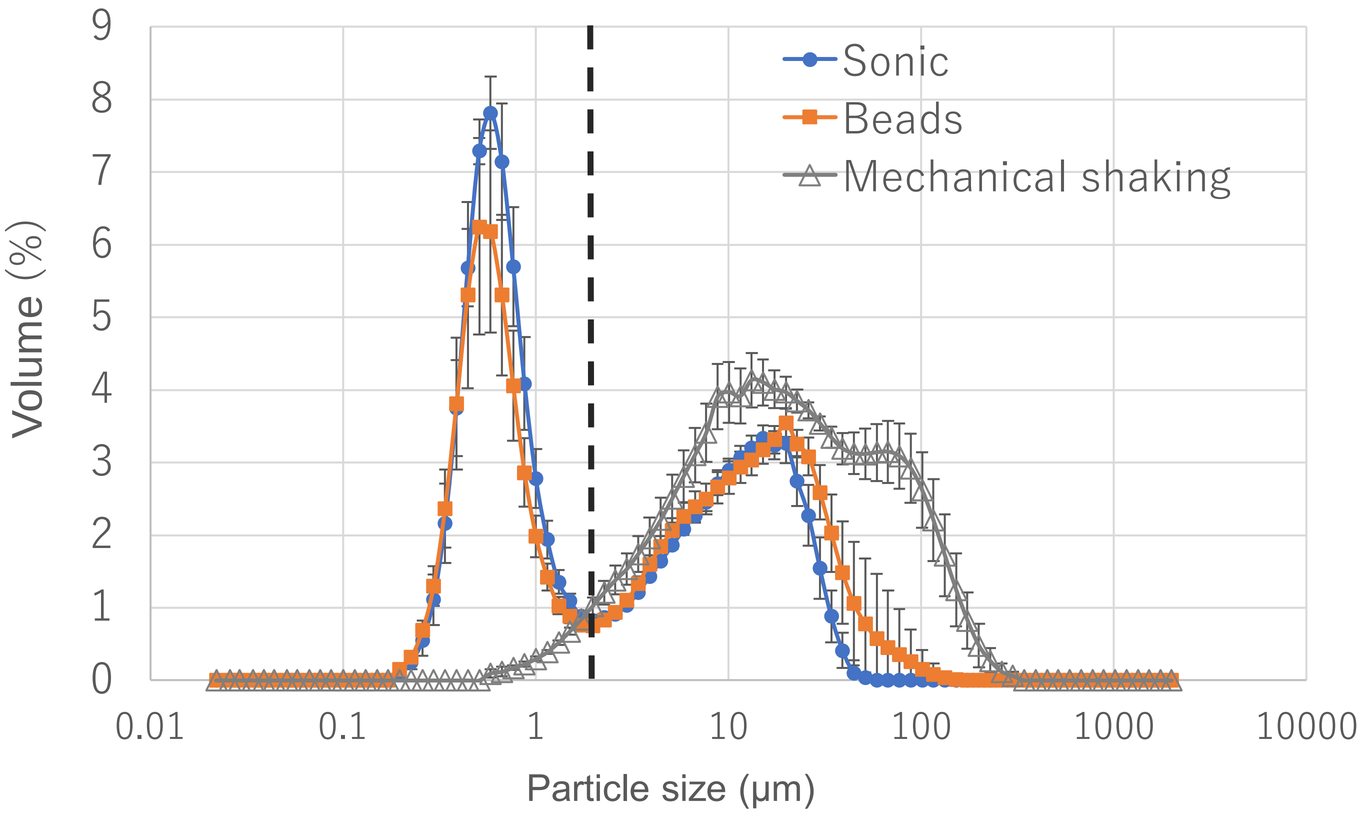

Fig. S2 Particle size distribution of single soil aggregates after the sonication, beads-vortexing and mechanical shaking dispersion treatments (n=3). The bimodal distribution boundary at 1.98 mm was shown as a vertical dotted line.

Fig. S3

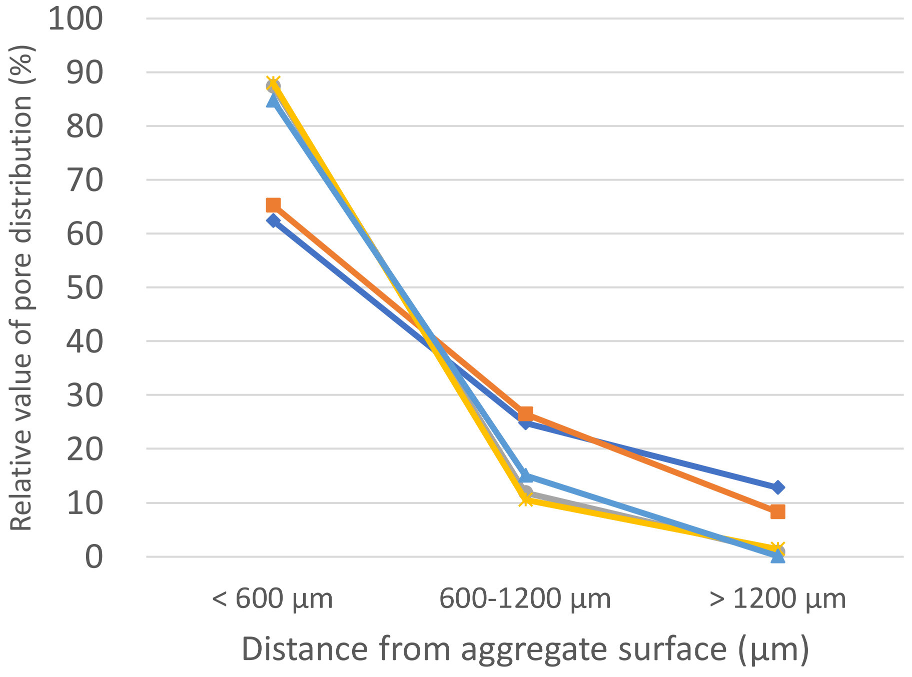

Fig. S3 Pore depth distribution of water-stable macro aggregate by X-ray µCT analysis. The different aggregates are shown by different colors and symbols.

Fig. S4

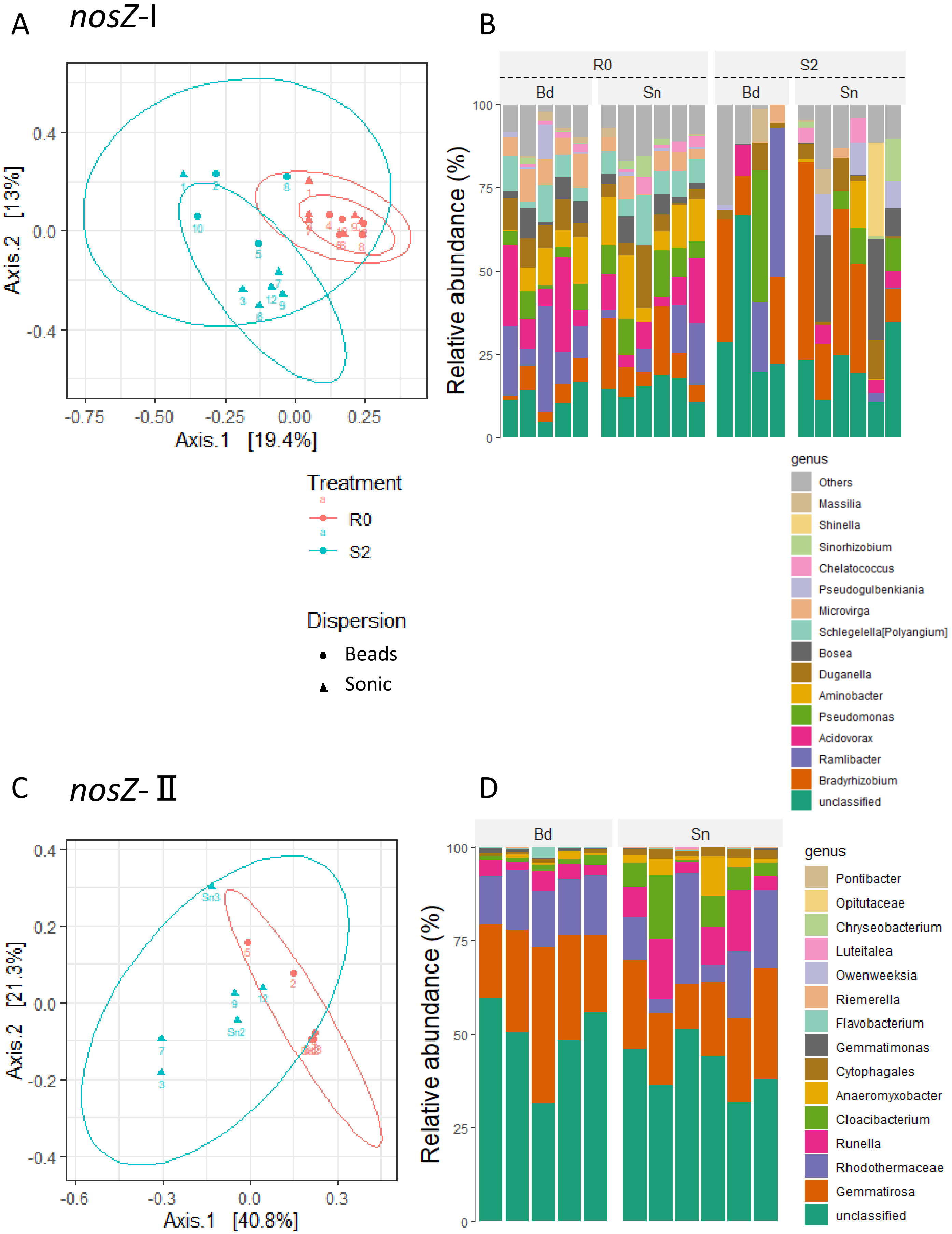

Fig. S4 Community structure by *nosZ*-I (A and B) and -II (C and D) amplicon analysis. Community differences are shown by a PCoA plot of the weighted UniFrac distance matrix (A, C) and a bar chart at the genus level (B, D) across the different fractions(R0: residue, S2: second supernatant) and dispersion methods (Bd: bead-vortexing, Sn: sonication treatment). The numbers indicate the sample ID.

Fig. S5

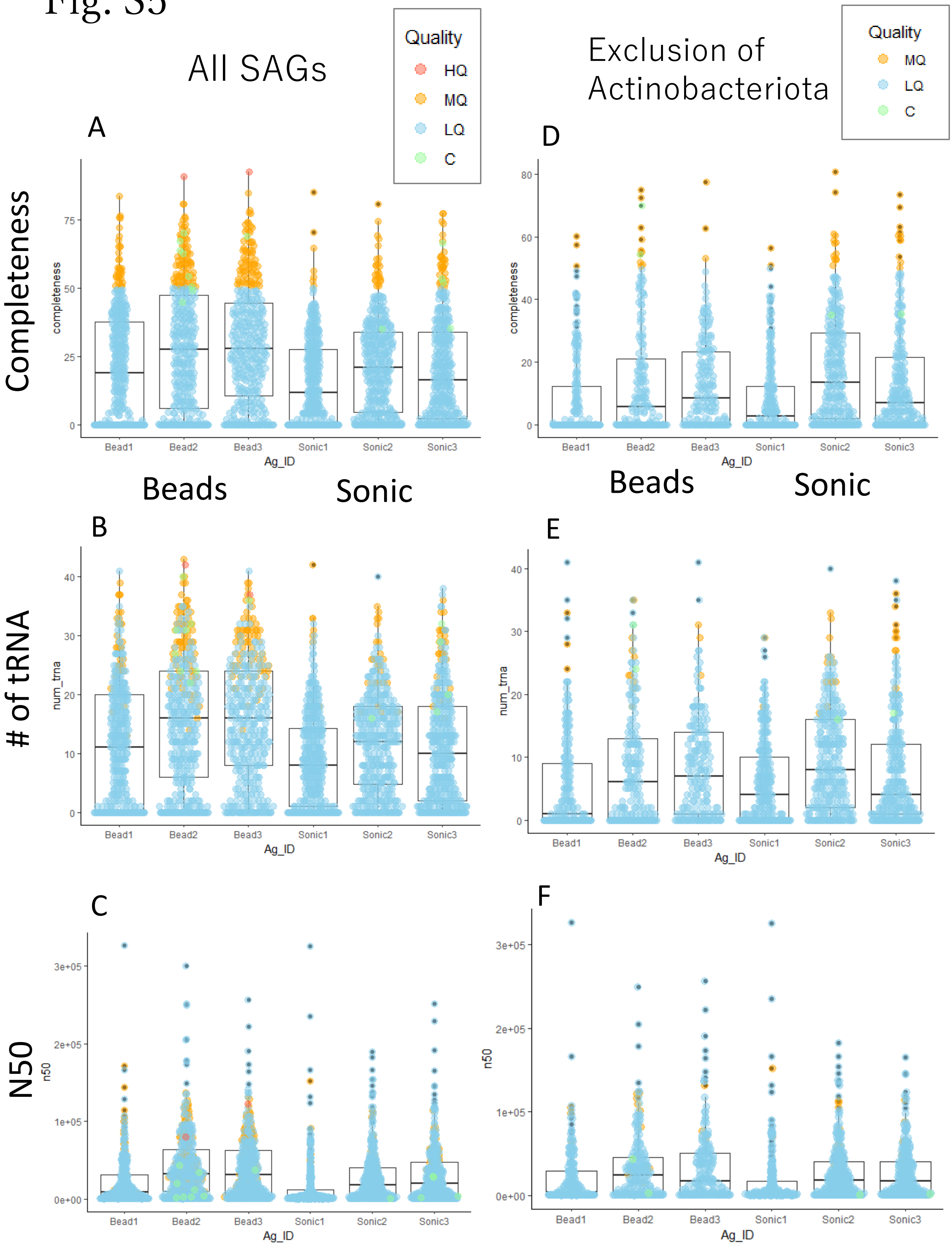

Fig. S5 Genomic quality of the SAGs. Data for all SAGs in A, B and C. Data for exclusion of Actinobacteriota in D, E and F. A and D indicates Completeness. D and E indicate number of tRNA. C and F indicate N50.

Fig. S6

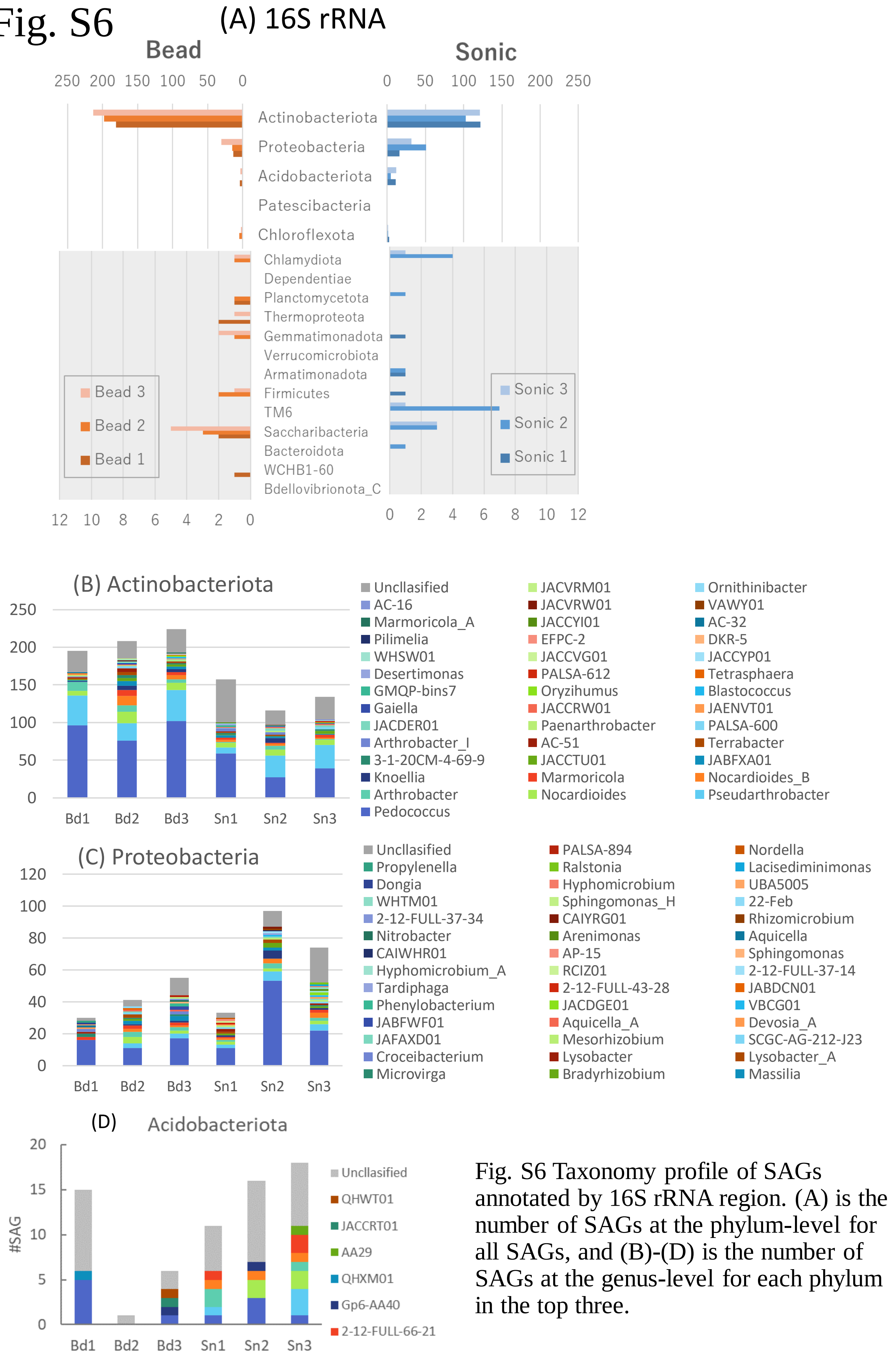

Fig. S6 Taxonomy profile of SAGs annotated by 16S rRNA region. (A) is the number of SAGs at the phylum-level for all SAGs, and (B)-(D) is the number of SAGs at the genus-level for each phylum in the top three.

Fig. S7

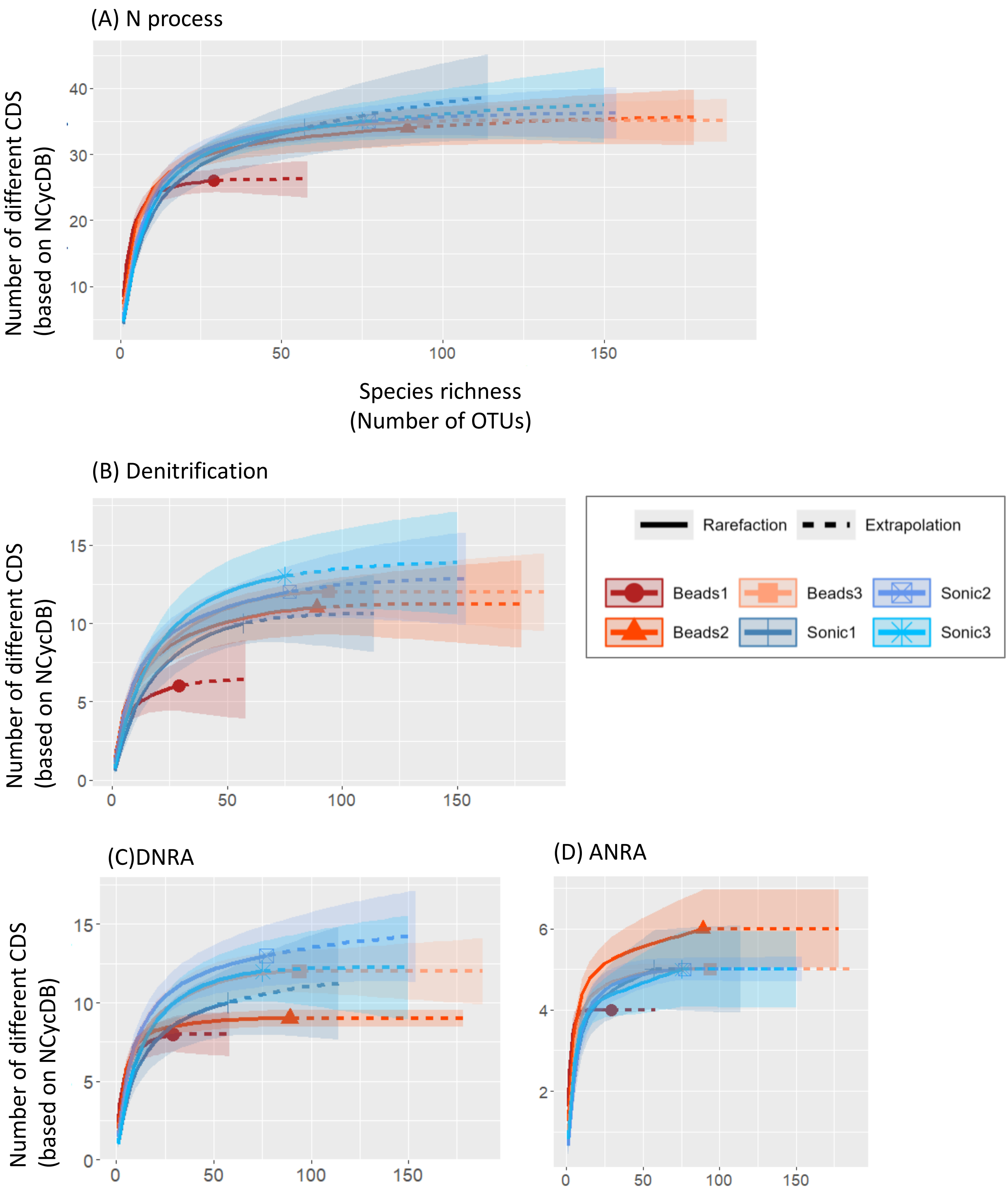

Fig. S7 Rarefaction curve of CDS related to N-cycling against number of OTUs in single aggregate.

Fig. S8

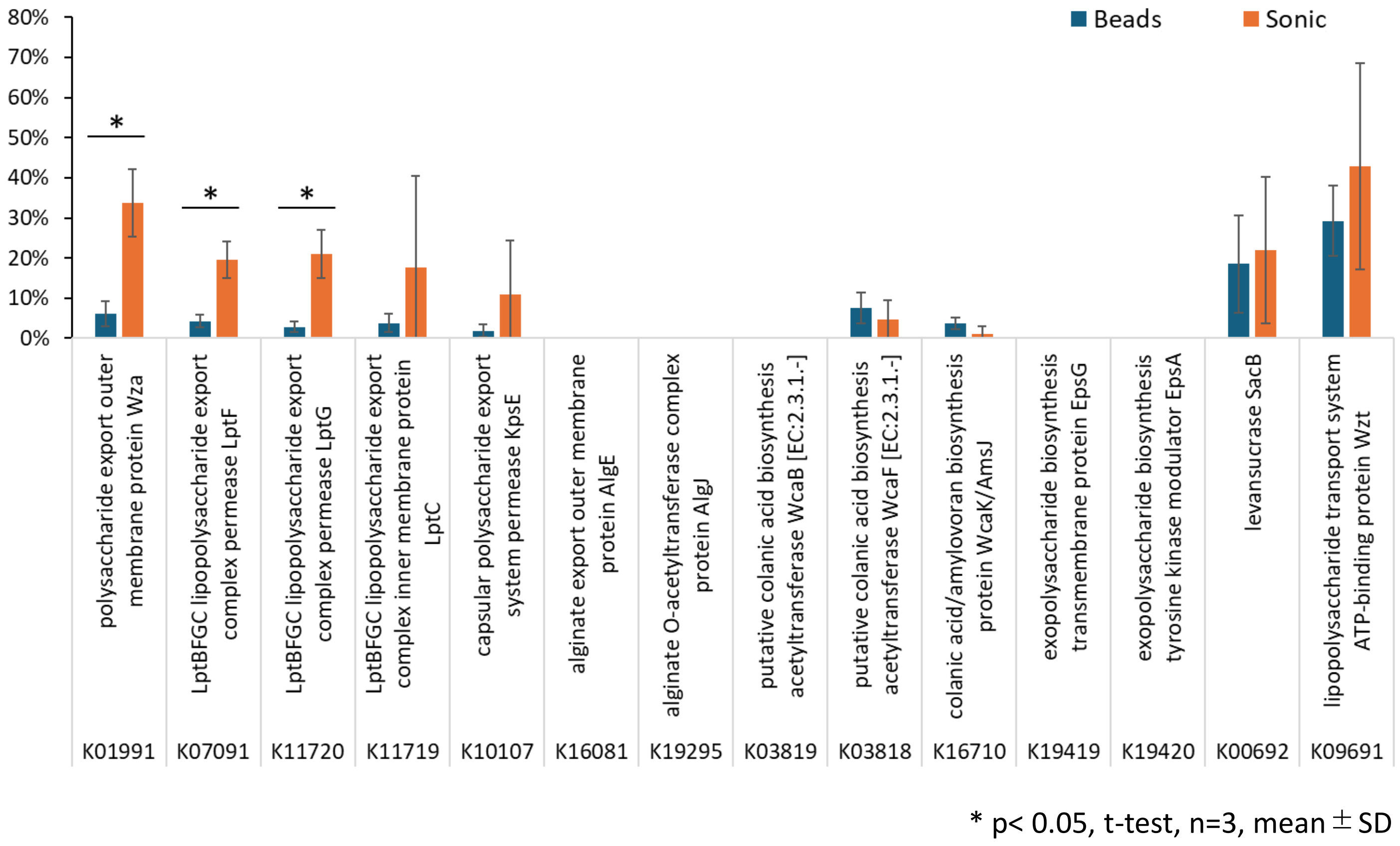

Fig. S8 The proportion of SAGs harboring the EPS related genes

Fig. S9

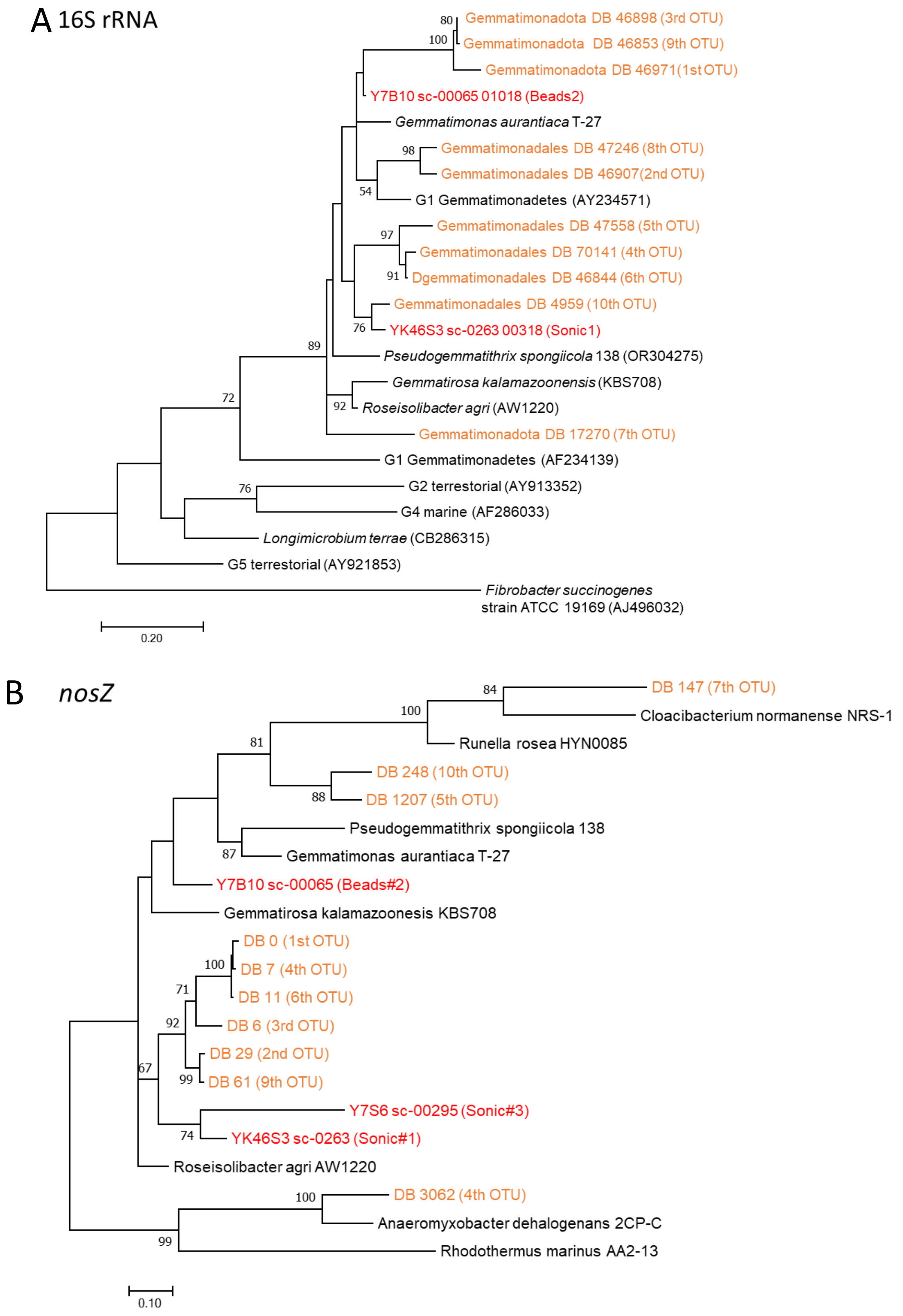

Fig. S9 Molecular phylogenetic analysis by Maximum likelihood method. A phylogenetic tree of 16S rRNA within Gemmatimonadota and *nosZ* was constructed using the top ten OTUs (≥99% sequence identity) in *nosZ*II amplicon analysis of the supernatant (orange color) , sequences of SAGs (red color) and reference sequences (black color). The 16S rRNA of *Fibrobacter succinogenes* strain ATCC 19169 (accession number AJ496032) as an outgroup in the phylogenetic tree (A). Bold number that support a probability >50% in bootstrap analyses (based on 500 replicates) are shown.

Fig. S10

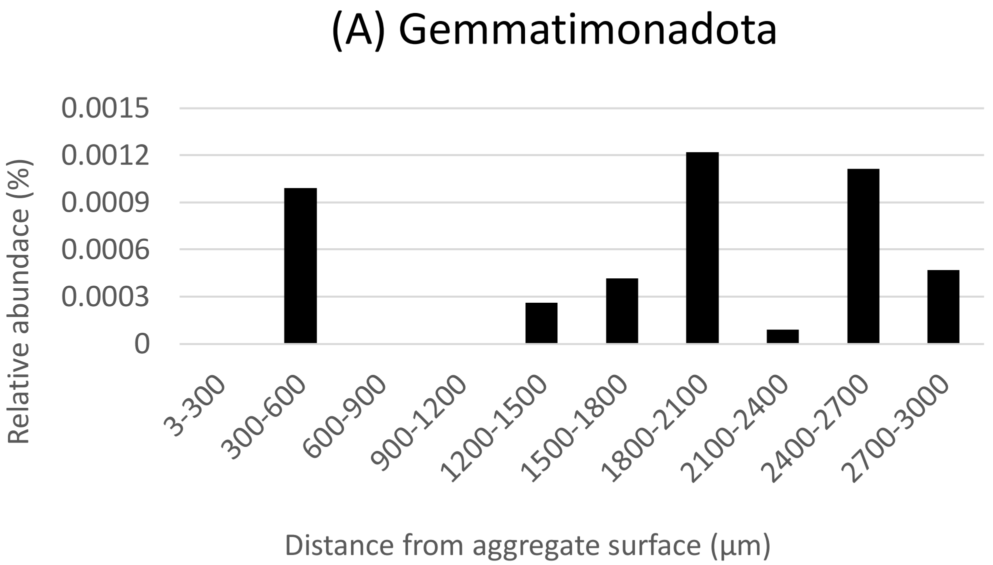

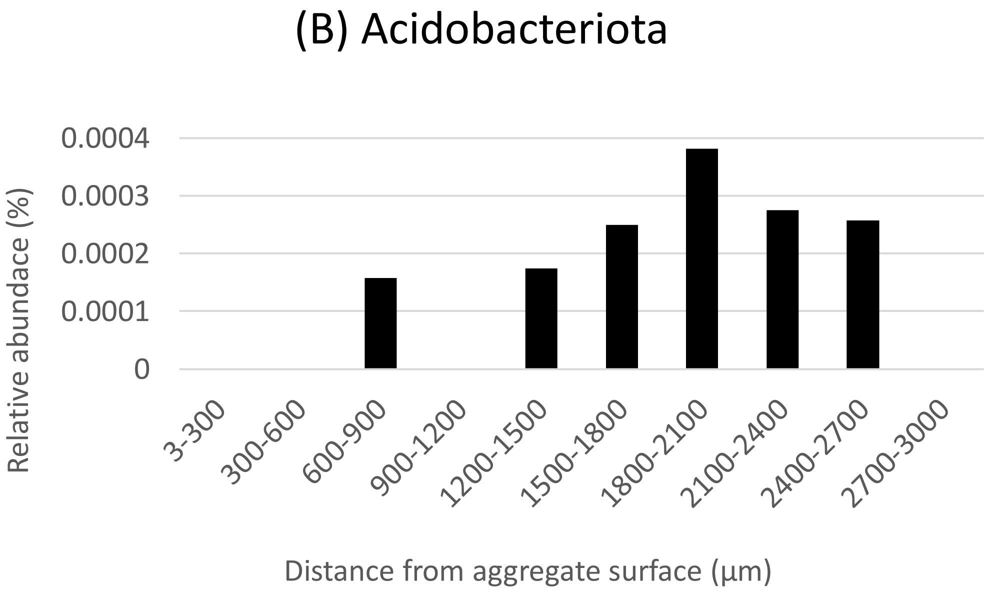

Fig. S10 The depth variations of OTUs with 99-100% homology to the *nosZ* of SAGs in the previous study (Mitsunobu et al. 2025).

Fig.S11

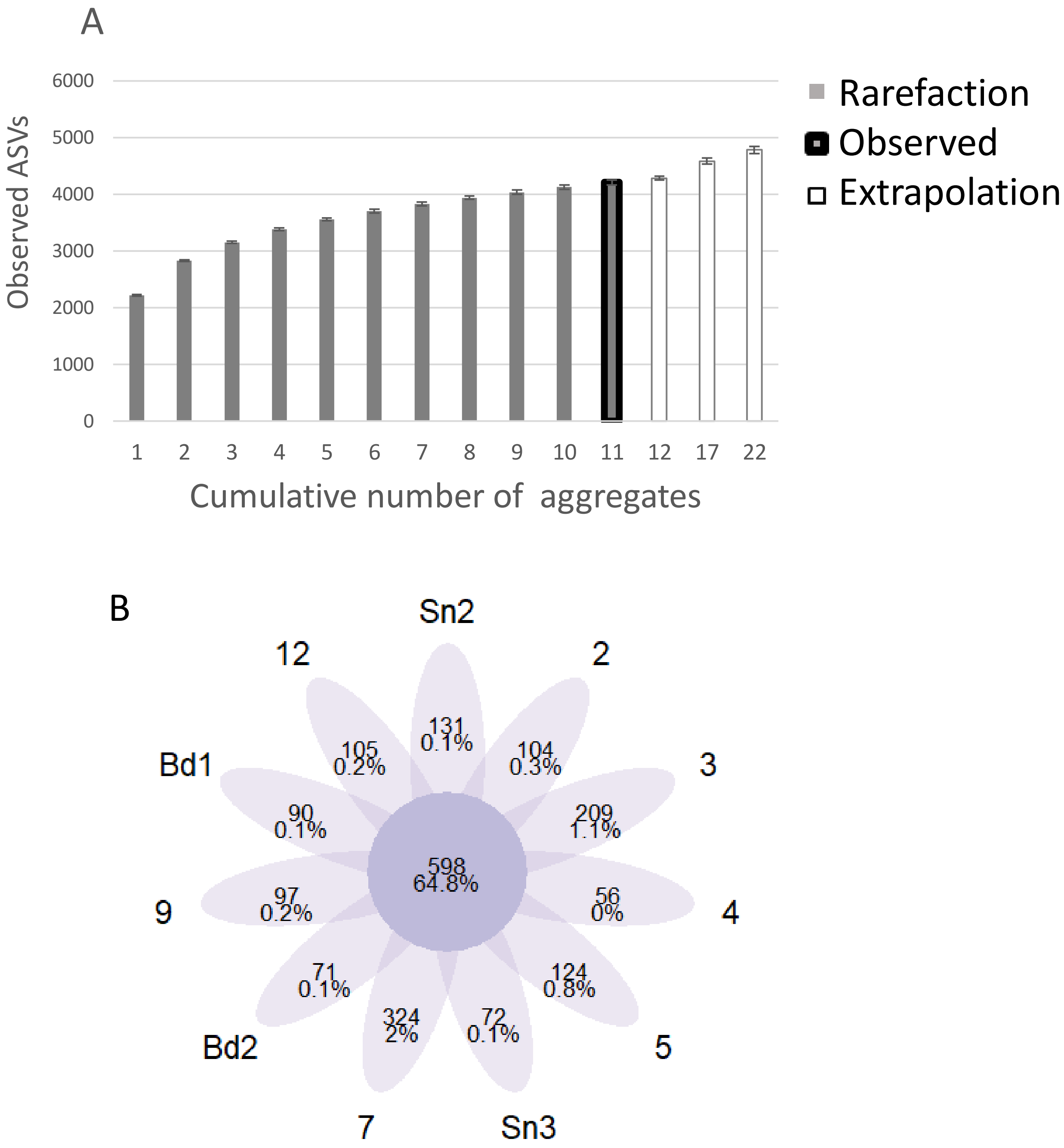

Fig.S11 Uniqueness and similarity of microbial communities of single aggregate. Species accumulation barplot (A) and venn diagram (B) were generated from ASV count tables combining R0 and S2 in each soil aggregate sample. Note that no data on S2 of aggregate #4 (missing values). The venn diagram indicated observed ASVs (the integer) and proportion of total abundances (%) of ASVs that were detected in all aggregates sample or detected exclusively only in specific single aggregate.

Table S1 Soil sample data and microbial data for each aggregate.

							DNA	Copy number					
								Sample			Per soil d. w. g		
Dispersion	Fraction	Aggegate ID	Soil weight dry	Soil weight wet	Diameter	Volume	Total DNA (µg)	16S rRNA	nosZ clade I	nosZ clade II	16S rRNA	nosZ clade I	nosZ clade II
Sonic	R0	Sn2	0.121	0.143	6.48	111.3	0.090	7.5E+06	1.2E+06	1.5E+04	6.2E+07	9.9E+06	1.2E+05
Beads	R0	2	0.080	0.097	6.97	145.0	0.188	3.7E+06	1.2E+06	3.2E+04	4.6E+07	1.5E+07	4.0E+05
Sonic	R0	3	0.054	0.066	6.13	98.4	0.074	1.3E+07	1.3E+06	1.0E+05	2.4E+08	2.4E+07	1.9E+06
Beads	R0	4	0.105	0.128	6.34	100.0	0.202	1.0E+07	1.8E+06	1.1E+05	9.5E+07	1.7E+07	1.1E+06
Beads	R0	5	0.055	0.067	5.78	77.3	0.043	4.1E+06	1.5E+06	2.9E+04	7.4E+07	2.7E+07	5.3E+05
Sonic	R0	Sn3	0.189	0.236	7.46	187.1	0.102	3.9E+06	1.5E+06	2.4E+04	2.1E+07	8.2E+06	1.2E+05
Sonic	R0	7	0.052	0.068	5.71	71.3	0.100	4.4E+06	1.5E+06	6.8E+03	8.4E+07	2.9E+07	1.3E+05
Beads	R0	Bd3	0.168	0.206	8.21	190.7	0.388	4.0E+07	1.6E+06	1.2E+06	2.4E+08	9.7E+06	7.4E+06
Sonic	R0	9	0.095	0.112	5.70	92.8	0.067	1.1E+07	1.3E+06	1.7E+05	1.1E+08	1.4E+07	1.8E+06
Beads	R0	Bd2	0.081	0.102	5.20	82.9	0.298	5.1E+07	1.3E+06	9.2E+05	6.3E+08	1.6E+07	1.1E+07
Sonic	R0	12	0.062	0.073	6.25	100.6	0.082	6.6E+06	1.6E+06	1.9E+04	1.1E+08	2.5E+07	3.1E+05
Sonic	S2	Sn2						7.8E+05	7.8E+04	3.0E+03	6.5E+06	6.5E+05	2.5E+04
Beads	S2	2						3.5E+05	3.9E+04	5.4E+03	4.4E+06	4.8E+05	6.7E+04
Sonic	S2	3						2.1E+05	3.4E+04	4.2E+03	3.9E+06	6.4E+05	7.9E+04
Beads	S2	4						ND	ND	ND	ND	ND	ND
Beads	S2	5						1.0E+05	3.0E+04	9.8E+02	1.8E+06	5.4E+05	1.8E+04
Sonic	S2	Sn3						5.3E+05	1.1E+05	3.8E+03	2.8E+06	5.9E+05	2.0E+04
Sonic	S2	7						1.8E+05	7.7E+04	1.3E+03	3.4E+06	1.5E+06	2.4E+04
Beads	S2	Bd3						2.6E+05	6.2E+04	8.1E+02	1.6E+06	3.7E+05	4.8E+03
Sonic	S2	9						4.6E+05	3.6E+04	2.7E+03	4.8E+06	3.7E+05	2.8E+04
Beads	S2	Bd2						2.8E+05	7.3E+04	9.2E+02	3.5E+06	9.0E+05	1.1E+04
Sonic	S2	12						3.3E+05	2.9E+04	2.5E+03	5.3E+06	4.7E+05	4.0E+04
Sonic	ND	Bd1	0.056	0.074	5.57	69.2	ND	ND	ND	ND	ND	ND	ND
Beads	ND	Sn1	0.074	0.098	6.12	77.9	ND	ND	ND	ND	ND	ND	ND

		α - diversity in 16S rRNA				
Dispersion	Fraction	Aggegate ID	Observed	Chao1	PD	Shannon	InvSimpson
Sonic	R0	Sn2	2241	2863	3290	6.15	123.8
Beads	R0	2	1952	2409	3038	5.78	78.7
Sonic	R0	3	2201	2865	3248	6.09	101.8
Beads	R0	4	2218	2794	3265	6.16	135.1
Beads	R0	5	1301	1583	2402	5.71	98.2
Sonic	R0	Sn3	1972	2286	3073	6.09	99.6
Sonic	R0	7	2189	2650	3255	6.06	85.7
Beads	R0	Bd3	2336	2893	3383	6.38	165.0
Sonic	R0	9	2195	2750	3266	6.24	147.2
Beads	R0	Bd2	2242	2803	3315	6.33	192.5
Sonic	R0	12	2184	2760	3258	5.94	102.7
Sonic	S2	Sn2	1345	1462	2429	5.46	56.2
Beads	S2	2	810	849	1778	4.89	27.7
Sonic	S2	3	760	922	1709	5.96	194.2
Beads	S2	4	ND	ND	ND	ND	ND
Beads	S2	5	591	651	1442	4.83	21.2
Sonic	S2	Sn3	1117	1213	2191	5.44	60.3
Sonic	S2	7	696	746	1645	6.18	314.1
Beads	S2	Bd3	881	918	1890	4.47	19.1
Sonic	S2	9	1090	1161	2159	5.47	49.4
Beads	S2	Bd2	866	920	1872	4.59	17.2
Sonic	S2	12	1074	1160	2128	5.54	69.4
Sonic	ND	Bd1	ND	ND	ND	ND	ND
Beads	ND	Sn1	ND	ND	ND	ND	ND

Table S2 The biomarker that characterizes the dissimilarity of the community at the phylum level among treatments by LEfSe analysis."Group" includes the residue after the beads treatment (R0Bd) and the sonication treatment (R0Sn), the supernatant after the beads treatment (S2Bd) and the sonication treatment (S2Sn).

Domain	Phylum	Group	LDA	P.unadj	P.adj	Significance
Bacteria	Chloroflexota	R0Bd	3.625	0.008	_0.034_	*
Bacteria	Proteobacteria	R0Bd	5.243	0.001	_0.014_	*
Bacteria	Nitrospirota	R0Bd	3.569	0.019	0.056	
Archaea	Euryarchaeota	R0Sn	2.323	0.002	_0.016_	*
Bacteria	Fibrobacterota	R0Sn	2.974	0.000	_0.014_	*
Bacteria	Firmicutes	R0Sn	2.859	0.001	_0.014_	*
Bacteria	Actinobacteriota	S2Bd	5.289	0.010	_0.037_	*
Bacteria	Parcubacteria	S2Bd	3.390	0.011	_0.040_	*
Bacteria	Armatimonadota	S2Sn	3.338	0.005	_0.027_	*
Bacteria	Bacteroidota	S2Sn	3.315	0.004	_0.022_	*
Bacteria	Candidatus_Saccharibacteria	S2Sn	4.409	0.001	_0.014_	*
Bacteria	Gemmatimonadota	S2Sn	3.876	0.003	_0.018_	*
Bacteria	Chlamydiota	S2Sn	3.615	0.026	0.070	
Bacteria	Candidatus Latescibacterota	S2Sn	2.823	0.037	0.090	
